# Incorporation of Low Concentrations of Gold Nanoparticles: Complex Effects on Radiation Response and Fate of Cancer Cells

**DOI:** 10.3390/pharmaceutics14010166

**Published:** 2022-01-11

**Authors:** Lucie Dobešová, Theresa Gier, Olga Kopečná, Eva Pagáčová, Tomáš Vičar, Felix Bestvater, Jiří Toufar, Alena Bačíková, Pavel Kopel, Radek Fedr, Georg Hildenbrand, Iva Falková, Martin Falk, Michael Hausmann

**Affiliations:** 1 Institute of Biophysics, The Czech Academy of Sciences, 612 65 Brno, Czech Republic; dobesova.lucy@ibp.cz (L.D.); kopecna@ibp.cz (O.K.); pagacova@ibp.cz (E.P.); toufar.jiri@gmail.com (J.T.); alenab@ibp.cz (A.B.); fedr@ibp.cz (R.F.); ivafalk@ibp.cz (I.F.); 2 Faculty of Science, Masaryk University, 611 37 Brno, Czech Republic; 3 Kirchhoff Institute for Physics, Heidelberg University, 69120 Heidelberg, Germany; theresa@fam-gier.de (T.G.); hilden@kip.uni-heidelberg.de (G.H.); 4 Department of Biomedical Engineering, Faculty of Electrical Engineering and Communication, Brno University of Technology, 616 00 Brno, Czech Republic; tomasvicar@gmail.com; 5 German Cancer Research Center (DKFZ), 69120 Heidelberg, Germany; f.bestvater@dkfz.de; 6 Department of Inorganic Chemistry, Faculty of Science, Palacky University Olomouc, 779 00 Olomouc, Czech Republic; pavel.kopel@upol.cz

**Keywords:** gold nanoparticles (GNP), nanoparticle-enhanced cancer radiotherapy, DNA double strand breaks (DSBs), DNA repair, chromatin nano-architecture rearrangements, single-molecule localization microscopy (SMLM), ionizing radiation-induced (repair) foci (IRIF), DNA repair nano-clusters

## Abstract

**(1) Background**: In oncology research, a long-standing discussion exists about pros and cons of metal nanoparticle-enhanced radiotherapy and real mechanisms behind the tumor cell response to irradiation (IR) in presence of gold nanoparticles (GNPs). A better understanding of this response is, however, necessary to develop more efficient and safety nanoparticle (NP) types designed to disturb specific processes in tumor cells. **(2) Aims and Methods**: We combined 3D confocal microscopy and super-resolution single molecule localization microscopy (SMLM) to analyze, at the multiscale, the early and late effects of 10 nm-GNPs on DNA double strand break (DSB) induction and repair in tumor cells exposed to different doses of photonic low-LET (linear energy transfer) radiation. The results were correlated to different aspects of short and long-term cell viability. SkBr3 breast cancer cells (selected for the highest incidence of this cancer type among all cancers in women, and because most breast tumors are treated with IR) were incubated with low concentrations of GNPs and irradiated with ^60^Co γ-rays or 6 MV X-rays. In numerous post-irradiation (PI) times, ranging from 0.5 to 24 h PI, the cells were spatially (3D) fixed and labeled with specific antibodies against γH2AX, 53BP1 and H3K9me3. The extent of DSB induction, multi-parametric micro- and nano-morphology of γH2AX and 53BP1 repair foci, DSB repair kinetics, persistence of unrepaired DSBs, nanoscale clustering of γH2AX and nanoscale (hetero)chromatin re-organization were measured by means of the mentioned microscopy techniques in dependence of radiation dose and GNP concentration. **(3) Results**: The number of γH2AX/53BP1 signals increased after IR and an additional increase was observed in GNP-treated (GNP(+)) cells compared to untreated controls. However, this phenomenon reflected slight expansion of the G2-phase cell subpopulation in irradiated GNP(+) specimens instead of enhanced DNA damage induction by GNPs. This statement is further supported by some micro- and nano-morphological parameters of γH2AX/53BP1 foci, which slightly differed for cells irradiated in absence or presence of GNPs. At the nanoscale, Ripley’s distance frequency analysis of SMLM signal coordinate matrices also revealed relaxation of heterochromatin (H3K9me3) clusters upon IR. These changes were more prominent in presence of GNPs. The slight expansion of radiosensitive G2 cells correlated with mostly insignificant but systematic decrease in post-irradiation survival of GNP(+) cells. Interestingly, low GNP concentrations accelerated DSB repair kinetics; however, the numbers of persistent γH2AX/53BP1 repair foci were slightly increased in GNP(+) cells. **(4) Conclusions**: Low concentrations of 10-nm GNPs enhanced the G2/M cell cycle arrest and the proportion of radiosensitive G2 cells, but not the extent of DNA damage induction. GNPs also accelerated DSB repair kinetics and slightly increased presence of unrepaired γH2AX/53BP1 foci at 24 h PI. GNP-mediated cell effects correlated with slight radiosensitization of GNP(+) specimens, significant only for the highest radiation dose tested (4 Gy).

## 1. Introduction

Cancer patients are often treated with radiotherapy [1], which, besides surgery, has been very effective and successful thus far. The reason for the success depends on a higher sensitivity to DNA damage of fast proliferating cancer cells compared to slowly proliferating normal cells. Moreover, cancer cells accumulate genomic alterations and become genetically unstable, which makes them further vulnerable to DNA damaging agents, including ionizing radiation (IR). The therapeutic window in radiotherapy is thus given by the radiosensitivity difference between the cancer and normal cells. This window may be very narrow or not exist at all as the tumor cells can often compensate their defects in DNA repair by an enhanced tolerance to unrepaired damage and become highly radioresistant. Hence, especially for tumors located in close proximity to organs or tissues with vital functions, the requirements on the precise radiation dose delivery and selectivity of radiotherapy can critically co-determine the treatment strategies and success. The selectivity of radiation damage delivery to tumor cells has been dramatically improved by a continuous development of irradiation techniques and, in some cases, by application of protons [2] or high-LET particle radiation [3,4] (comprehensively reviewed in [5]). However, these improvements are still insufficient for many tumors. This prevents full irradiation of tumor edges and leads to incomplete treatment outputs associated with selection of radio-resistant tumor cell clones. Hence, an urgent need persists for a therapeutic strategy that will enhance the radiation-induced damage to the tumor cells but, at the same time, preserve the normal tissues in the tumor surroundings as much as possible.

A newly proposed approach to further selectively potentiate radiation sensitivity of tumors is based on incorporation of metal nanoparticles (NPs) into the tumor tissue or tumor cells prior to irradiation [6,7,8,9,10,11,12,13,14] (comprehensively reviewed in [5]). The original idea behind this selective radiosensitization is that, due to high electron content and high photoelectric absorption of high-Z materials (Z = atomic number of the element), metal NPs emit showers of secondary electrons upon irradiation [15,16]. The ejected electrons can directly damage DNA through dissociative electron attachments [17] and generate microscale clouds of ionizations leading to water radiolysis and production of reactive oxygen species (ROS) [18,19] concentrated around nanoparticle location sites. Irradiation thus amplifies radiation dose within limited cell volumes [18], which leads to locally enhanced radiation damage followed by more efficient tumor cell killing [20,21]. NPs can also generate ROS by themselves, i.e., in absence of radiation, which has been related to their cytotoxicity [19]. In addition to DNA damage, increased ROS in the cytoplasm can also react with other main biomolecule types—lipids and proteins—damage various cell organelles, and initiate cell death via numerous mechanisms [19]. This fact suggests that NP-mediated cell radiosensitization can by no means be completely explained by a simple production of secondary electrons [19], as discussed later.

As the normal cells internalize NPs less efficiently than the tumor cells [22], this enhanced damaging is mostly directed to the cancer cells, thus providing the normal cells with a higher opportunity to survive. In other words, for the tumors responding well to irradiation, the macroscopic (therapeutic) IR dose can be decreased in the presence of NPs compared to irradiation alone to better protect the normal tissues in the tumor surroundings with unchanged impact on the tumor. For the radioresistant tumors, on the other hand, a non-reduced dose can be applied to enhance damage to the tumor with preserved protection of the normal tissues. Moreover, as GNPs are internalized by tumor cells even passively and therefore enriched in tumors [22,23,24,25,26,27], they can be simultaneously used for tumor radiosensitization and precise imaging [28] for monitoring of radiotherapy procedures (theranostics) [29]. In addition, GNPs provide a large room for surface functionalization, which allows their targeting to specific cellular organelles, such as the endoplasmic reticulum [14], or association with various (anti-cancer) drugs [30].

The radiosensitizing effects of metal NPs were experimentally confirmed both in cells [13,14,31,32] and animal models [33]. For numerous cell types, an increased cell death was observed when GNPs were added to the cell culture several hours prior to radiation exposure [6,34,35]. However, the effort to repeat these findings for some other NP and/or cell types has failed [32], pointing to our very inconclusive understanding of biological effects provoked by irradiated GNPs and the mechanisms responsible for these effects [13].

Chromatin is the main target for IR as the nuclear DNA is highly sensitive to radiation damage and, in contrast to other biomolecules, cannot be simply replaced if the original (epi)genetic information has been lost due to imprecise repair [36,37,38,39]. The most severe damaging effect of IR is the fragmentation of nuclear chromatin by single-stranded (SSB) and double-stranded (DSBs) DNA breaks (for review [40]). It is therefore assumed that the physical mechanism of cell radiosensitization by metal (high-Z) NPs is based on the physical enhancement of SSB and DSB induction in nuclear DNA [18]. In accordance, GNPs and other metal NPs have been shown to increase the numbers of radiation-induced SSBs and DSBs in isolated DNA plasmids [18,34]. Moreover, the clouds of ionization events concentrated around NP location sites could be expected to increase not only the numbers of DSBs but also their multiplicity and/or complexity [41]. These multiple/complex lesions can hardly be repaired completely [41,42,43,44] and are therefore the main cause of the therapeutic efficiency of IR, especially of high-LET (linear energy transfer) radiation types with high radiobiological effectiveness (RBE). Based on this theoretical knowledge and experiments proving radiation damage enhancement on in vitro systems [18,34], it is reasonable to hypothesize that NPs deliver the radiosensitization effect by the same mechanism also in cells. Some research groups indeed demonstrated increased DNA break numbers in the nuclear DNA of intact cells, both after γ-ray and heavy ion irradiation in presence of NPs [32,34] (see also references in [6,13]); however, the excessive damage was usually only weak or moderate, while other studies failed to observe this phenomenon completely (e.g., [6], for a review see [13,32]). These results have raised the question whether the excessive DNA damage, if it appears, could be sufficient to ensure the observed cell killing, sometimes enhanced by a factor of 1.5 or even higher (e.g., [45]). Noticeably, some studies reported more extensive DNA radiation breakage in the presence of NPs without being accompanied by the corresponding changes of the dose–response (cell survival) curve [32].

The main problem with the “nucleocentric” hypothesis of the radiosensitization is the cytoplasmic localization of NPs within the cells. While metal NPs of sizes up to ~50 nm can cross the cell membrane and accumulate in the cytosol, even the smallest NPs are not able to penetrate the cell nucleus [6,14,28,46] unless they are modified for this purpose (tagged with DNA oligos) and transfected into cells by using a suitable DNA transfection protocol [6,31]. However, even in such cases, NPs primarily occupy the perinuclear space [14] and remain retained within the cytoplasm [31]. Hence, if only a very limited range of most secondary electrons is considered, a significant enhancement of radiation damage to the nuclear DNA by NPs can be questioned or explained in an alternative way. Moreover, the hypotheses of NP radiosensitization based on DNA damage enhancement are also challenged in general—the mitochondria, the only organelles in human cells that contain DNA except for the cell nucleus, usually do not colocalize with NPs, though both mitochondria and NPs are located in the cytoplasm [32]. The biological processes responsible for nanoparticle-mediated (tumor) cell radiosensitization thus remain a subject of debate.

This uncertainty is largely due to enormous variability of NPs, cell types, radiation types, and other experimental conditions used, frequently leading to contradictory results. For this heterogeneity, a number of mechanisms have been postulated for the radiosensitization of cells by NPs, which must be further tested. The difficulties with interpreting the results of meta-analysis on GNP radiosensitization also stem from the fact that although the radiosensitization effect is always of concern, individual studies focus on different aspects of this phenomenon. For instance, some studies analyzed the effects of GNPs on the cell viability after radiation exposure, usually by the means of the clonogenic assay (also known as the colony forming assay, CFA) [31,32] or flow cytometric quantification of apoptosis. Other studies then evaluated DNA damage induction and/or repair [6,13,15], mostly by quantifying γH2AX foci [14,47] which have been considered a specific marker of DSBs [48] and currently provide the only opportunity to detect relatively low DSB numbers generated by clinically relevant radiation doses in intact cells [49]. The heterogeneity of experimental approaches and their incomparable or even contradictory results described above motivated us to perform a comprehensive study on GNP-mediated radiosensitization and explore, under given experimental conditions, the relationship between DSB induction, DSB repair and cell survival upon irradiation.

The relative dose-dependency of repair focus numbers does not seem to be different for cells of different radiation sensitivity; however, the intensity [50], topology [42,51,52,53] formation kinetics [42] and persistence [54] of various (γH2AX, 53BP1, MRE11) foci may differ among the cell types. Hence, in addition to simple evaluation of DSB induction and repair kinetics, a detailed multiparameter analysis was performed to compare the morphology and other features (e.g., intensity and mutual colocalization) of γH2AX and 53BP1 repair foci in cells irradiated in the presence or absence of GNPs. This analysis has been expected to help identify the character and nature [41,55] of the excessive DSBs, which, according to some studies, occur in cells irradiated with (G)NPs. The obtained knowledge may then explain why the enhanced DNA damage, even if observed, is not always correlated to a decreased post-irradiation cell survival.

Recent developments in the field of super-resolution localization microscopy [56,57] have made it possible to study the composition and topology of γH2AX and other repair protein clusters [53,58,59] even at the molecular (nano)scale [13,32]. As a unique aspect of this manuscript, various parameters of repair foci formation and DSB repair were addressed in parallel at the micro- and nanoscale, using high-resolution immunofluorescence confocal microscopy and single molecule localization microscopy (SMLM), respectively. SMLM has been recognized as especially useful for the purpose of the present study as it enables topological comparison of various molecular distributions and structures at the molecular level without a need for complicated and often misleading image analysis [56,58,59,60,61]. Various mathematical algorithms can directly be applied on the coordinate data matrices generated by SMLM (the fundamental concept of SMLM and topological analysis procedures have been described elsewhere [51,59,62,63] and in Methods) to calculate spatial distances and topological arrangements of the molecules with ~10 nm precision. Application of SMLM thus allow for comparing even subtle differences between chromatin architecture in SkBr3 cells [56] irradiated in the presence or absence of GNPs. As chromatin architecture and its changes have been shown to depend both on the cell status and irradiation conditions (dose and type) [56,62], we analyzed the architecture of heterochromatin domains (labeled with anti-H3K9me3 antibody) as another marker of radiosensitizing or cytotoxic GNP effects. The same procedure of γH2AX/53BP1 repair focus labeling and sample preparation for confocal microscopy and SMLM allows for correlative analysis of DSB repair foci at different spatial scales; still overlooked features of repair foci and thus excessive DSBs generated by radiation in the presence of NP may be revealed [41,55,56,58,60].

## 2. Materials and Methods

### 2.1. Cells and Cell Culturing

SkBr3 breast carcinoma cells [64] were chosen as the model because of the highest incidence of this type of tumor in women, and because most breast tumors are treated with radiotherapy [65]. In addition, SkBr3 cells with Her2/neu upregulation are a well-established model for radiation treatment studies [66]. SkBr3 cells also dispose with a high level of thioredoxin reductase [17,67], which can reduce penetration of ROS, produced by cytoplasmically located nanoparticles, into the cell nucleus [68,69]. This property is advantageous considering the scope of the present manuscript as it could be expected to help us to “separate” cytoplasmic and nuclear effects of nanoparticles.

The cells were cultured in McCoy 5A (SMLM experiments) or DMEM (remaining experiments), in both cases supplemented with 10% fetal calf serum and 1% penicillin/streptomycin. Cells in culture flasks were placed in the incubator set to 37 °C and 5% CO_2_ atmosphere.

### 2.2. Incubation of Cells with Gold Nanoparticles

Effects of 10-nm (diameter) gold nanoparticles (Aurion GoldSol 10 nm; cat. No. 410.011, AURION Immuno Gold Reagents & Accessories, Aurion, Wageningen, The Netherlands) on SkBr3 cells were studied in non-irradiated cells or cells exposed to different doses of photonic ionizing radiation, as described below. Before the nanoparticle treatment, the culture medium was completely removed from cell samples and replaced with the medium containing commercially available Aurion gold nanoparticles (GNP) of 10 nm in diameter. The stock GNP solution (1.5 × 10^12^ particles/mL) was diluted in the culture medium and added to cell samples to a final concentration of 0.4 or 0.8 μg/mL. After 18 h-incubation at 37 °C/5% CO_2_, the medium was exchanged by a fresh one, free of GNPs.

Commercially available GNPs were selected in the present manuscript to allow performance of all complex experiments with parametrically the same nanoparticles as much as possible, which is best ensured by commercially produced and controlled ones. However, at the same time, this brings together some limitations on the nanoparticle composition details. According to the manufacturer, the Aurion GNPs are uncoated, gold sols based on the strictly defined particle sizes of 10 nm. The sols are monodispersed, with a coefficient of variance <15%, but in most cases <10%. Surface mainly contains citrate molecules, as citrate is also used in preparation of the gold nanoparticles. The producer webpage is: https://aurion.nl/products/gold-nanoparticles/gold-sols/ (accessed on 25 November 2021). The product datasheet is available, e.g., from https://www.biotrend.com/file_fournisseur/0274_10bijsluiter-sol-18.1.pdf (accessed on 25 November 2021) but more detailed information is not available.

### 2.3. Irradiation

For SMLM, cell specimens with and without GNPs were irradiated in 6-well plates with a 6 MV linear accelerator (LINAC) radiation source without flattening filter. For the remaining analyses, a ^60^Co source (Chisostat, Chirana, Czech Republic) of *γ*-rays has been used. The cells were exposed to indicated radiation doses with a dose rate 1 Gy/min, at 37° C and in normal atmosphere. Either single doses of 0 (non-IR control), 2, 4 and 6 Gy or a fractionated dose of 2 Gy delivered in two 1 Gy fractions separated by 30 min (further referred to as 1 + 1 Gy dose) were studied.

### 2.4. Confocal Microscopy

#### 2.4.1. Sample Preparation and γH2AX/53BP1 Immunostaining

The cells were seeded on slides rinsed with 96% ethanol and placed into 4-well plates (Thermo Fisher Scientific, Rockford, IL, USA, cat. No. 167063). Four hours later, 4 *mL* of culture medium were added to each well. The second day of cultivation (5% CO_2_/37 °C), cell specimens were incubated with Aurion GNPs (0.4 μg/mL) as described in Section 2.2. After 18 h incubation (5% CO_2_/37 °C) the medium with GNPs was removed, and replaced with the fresh medium free of GNPs. Immediately after changing the medium the dishes were irradiated with a single dose of 2 Gy, or 4 Gy, or two doses of 1 Gy with 30 min time-out. After irradiation, the dishes were returned in an incubator (5% CO_2_/37 °C).

Cells were fixed in times 30 min, 4 h, 8 h and 24h post irradiation (PI) in 4% paraformaldehyde/PBS for 10 min at RT, and permeabilized with 0.2% Triton X-100/PBS for 15 min. A combination of two primary antibodies was used for the immunofluorescence detection: anti-phospho-Histone H2AX, clone JBW301 (mouse; Merck Millipore, Burlington, WA, USA, cat. No. 05-636-I, 1:300) + anti-53BP1 (rabbit; Cell Signaling Technology, Danvers, MA, USA, cat. No. 4937, 1:400). After the overnight incubation with primary antibodies (at 4 °C), a mixture of secondary antibodies FITC-conjugated donkey anti-mouse (Jackson Immuno Research Laboratories, West Grove, PA, USA, cat. No. 715-095-150, 1:100) and Cy3-conjugated donkey anti-rabbit (Jackson Immuno Research Laboratories, West Grove, PA, USA, cat. No. 711-165-152, 1:200) was applied for 1 h (RT). The cell nuclei were stained with DAPI (5 min at RT) at a dilution of 1:20.000 and mounted to Vectashield mounting medium (Vector, Burlingame, WA, USA, cat. No. H-1000).

#### 2.4.2. Immunofluorescence Confocal Microscopy

Confocal microscopy images of the immunofluorescent staining were captured using an automated high resolution Leica DM RXA microscope (Leica, Wetzlar, Germany) equipped as follows: a Plan Fluotar oil immersion objective (100x/NA1.3); a CSU 10a Nipkow disc (Yokogawa, Japan); a CoolSnap HQ CCD-camera (Photometrix, Tuscon, AZ, USA); and an Ar/Kr-laser (Innova 70C Spectrum, Coherent, Santa Clara, CA, USA). Acquiarium software [41] was used for automated image acquisition. Fifty serial optical sections were captured at 0.3-μm intervals along the z-axis. See [41] for detailed procedure descriptions.

##### 2.4.3. γH2AX/53BP1 Focus Detection, Scoring and Micro-Morphology Analysis

γH2AX and 53BP1 visualization for 3D focus modeling was performed in IMARIS 9.7 software package, Oxford Instruments, Oxford, UK (https://imaris.oxinst.com/; accessed on 5 January 2022). Image analysis was performed using custom MATLAB software [70], which consists of automatic 3D segmentation of individual nuclei and semi-automatic detection of ionizing radiation-induced (repair) foci (IRIFs) composed of γH2AX/53BP1, which was then used as seeds for segmentation of individual IRIFs. The resulting segmentation was used for the extraction of morphological and intensity base parameters of IRIFs of individual nuclei. The nuclei segmentation process includes segmentation with 3D convolutional neural network (CNN) with U-Net architecture [71]. This network produces binary segmentation; thus, it was followed by distance transform and watershed in order to separate touching nuclei into individual objects. For more details about this method and training, see [72].

Detection of IRIFs consists of automatic extraction of proposal points, which were then manually filtered by an expert in a graphical user interface. Proposal IRIFs were detected as local maxima in colocalization image, which was created as a multiplication of the two IRIF channels (pixel-wise multiplied image of γH2AX and 53BP1) [70]. Moreover, only local maxima on the foreground of Maximally Stable Extremal Regions (MSER) [70,73] segmentation were considered as proposal points. IRIF detection was then carried out by manual selection of proposal points in a 2D projection image. Segmentation of individual IRIFs was achieved with MSER, which was applied to the colocalization image. The IRIF segmentation produced by MSER was then combined with IRIF detection employing the seeded watershed transform. The seeded watershed transform [74] was applied on the colocalization image, where the output of the IRIF detection served as the seeds and MSER results served as the foreground mask.

After segmentation, several parameters were extracted for subsequent analysis, including the number of IRIFs per nucleus, fraction of nucleus volume occupied by IRIFs, normalized focus intensity and colocalization between γH2AX and 53BP1 foci (defined below). Indicated features were jointly compared among specimens using Principal Component Analysis (PCA) [75], where results were visualized using biplots [76], which display both samples and original variables in PCA space of reduced dimensionality. PCA biplots can be used, e.g., for interpretation of variable dependencies and inspection of separation of data classes in high dimensional space. PCA and biplot visualization was performed in MATLAB.

γH2AX foci colocalizing with 53BP1 repair protein foci were scored as DSBs. This strategy of using two independent DSB markers ensured the maximum precision of DSB identification. In addition, the colocalization of γH2AX with repair proteins also indicates that these DSBs have not yet been repaired, ensuring that persistent γH2AX foci quantified at 24 h PI are still unrepaired DSBs [37]. Only the colocalized foci were analyzed, where the extent of colocalization was determined by detection and segmentation in a colocalized image (created by the multiplication of the γH2AX and 53BP1 channels). Colocalization of γH2AX and 53BP1 was calculated as a correlation of γH2AX and 53BP1 of IRIF pixels (i.e., inner pixels of segmented IRIFs are used for each nucleus, where Pearson correlation is calculated between γH2AX and 53BP1 values of those pixels). The distribution of colocalization values between γH2AX and 53BP1 foci can be seen in Figure 5D in Section 3. As fluorescence intensities are very variable and cannot be quantified directly, extracted IRIF intensities are normalized, where values were divided by 99 percentiles of whole nuclei, and the average value for each IRIF was extracted.

Recently, we have shown [70] saturation of 53BP1 at short (0.5 h) post-irradiation periods of time at higher (≥4 Gy) radiation doses. However, this phenomenon is not relevant for the present analysis as the differences between GNP(−) and GNP(+) cells observed in the present manuscript reflect the proportion of cells with high γH2AX/53BP1 focus numbers and not the number of colocalized γH2AX and 53BP1 foci in cells.

### 2.5. SMLM

#### 2.5.1. γH2AX and Heterochromatin (H3K9me3) Immunostaining

The cells were transferred on the cover-slips and incubated until 40% confluence in 6-well plates. For SMLM labeling, the cells were permeabilized with 0.2% Triton-X100 in 1x PBS for 3 min, washed three times in 1x PBS for 5 min each and blocked with 2% BSA (bovine serum albumin) in 1x PBS. The cells were stained with rabbit anti-H3K9me3 antibodies (1:400, ab8898 Abcam, Cambridge, UK) and with mouse anti-γ-H2AX antibodies, clone JBW301 (1:250, cat. No: 05-636-I, Merck Chemicals) in a moist chamber at 37 °C for 30 min each. After three 5 min washes in PCTG (PBS, 0.1% Casein, 0.05% Tween20 and 0.5% fish gelatin (Sigma-Aldrich, Waltham, MA, USA), primary antibodies were detected with secondary goat anti-rabbit Alexa Fluor^®^488 (ab150077, Abcam, Cambridge, UK) and goat anti-mouse Alexa Fluor^®^568 (Abcam) antibodies for 45 min at 37 °C, followed by three 5 min washes in PCTG. Then the specimens were counterstained with DAPI and embedded in ProLong Gold anti-fade solution (Thermo Fisher Scientific, Waltham, MA, USA) for microscopic analysis.

##### 2.5.2. SMLM Data Acquisition and Analysis

The fundamental concept of SMLM and topological analysis procedures have been described elsewhere [51,52,56,59]. In brief, super-resolution is reached by optical isolation of fluorescently labeled individual molecules by switching between two different spectral states of the fluorophore, e.g., between the *on* and *off* state [77]. After illumination with a high-power laser, all fluorochrome molecules are switched to a so-called reversibly bleached dark state (the *off* state) but can afterwards randomly return to the bright photon emitting state (the *on* state) [78,79,80]. This fluorophore “blinking” ensures temporal isolation and thus spatial separation of individual fluorescent signals, which can be precisely localized by the barycenter of the Airy disc visualized by the microscope objective lens [57]. This allows the precise determination of 3D coordinates and additional parameters of each fluorochrome molecule and generation of a “pointillist” super-resolution image [81]. Most importantly, various mathematical algorithms can be directly applied on the coordinate data matrices to calculate spatial distances and topological arrangements of the molecules with ~10 nm precision [56]. Moreover, the labeling procedure is the same for SMLM and confocal microscopy, so the same samples can be correlatively analyzed at the micro- and nanoscale.

SMLM was performed on an in-house built localization microscope as described in detail in [52,81,82]. Enhanced thermo-mechanical stability and high precision optical elements led to a point localization precision of about ±10 nm. The illumination laser light path was equipped with a LightHub—laser combiner (Omicron Laserprodukte GmbH, Rodgau-Dudenhofen, Germany) for four laser lines (405 nm, 491 nm, 561 nm and 642 nm). These wavelengths were separated by a polychromatic AOTF (AA Opto Electronic, Orsay Cedex, France). The laser beams were shaped by a variable beam expander 10BE03-2-8 (Standa ltd, Vilnius, Lithuania) and a Flat-Top- Profile forming optics—PiShaper (AdlOptica GmbH, Berlin, Germany). The circular flat-top laser beam profile was focused into the object plane using an achromatic focusing lens (f = 250 mm) and a 100×/NA 1.46 oil plan apochromatic objective lens (Carl Zeiss Microscopy, Göttingen, Germany). The fluorescence light was separated from the illumination light by two quadband interference filter glasses F73-410 and F72-866 (AHF Analysentechnik AG, Tübingen, Germany). The emitted fluorescence light was magnified by the objective–tube lens pair (Carl Zeiss Microscopy, Göttingen, Germany) and a twofold expander before detected on an Andor Ultra EMCCD (iXonUltra 897, Andor Technology, Belfast, Northern Ireland) with a pixel size of 16 × 16 μm².

For each specimen and experimental condition, a minimum of 20 cells were recorded and evaluated. With 405 nm illumination, the widefield image of the DAPI staining was acquired. With 491 nm and 561 nm illumination, respectively, blinking of the Alexa dye molecules was induced and 2000 image frames were recorded for each color plane. Each image had an integration time of 100 ns.

Local positions of the detected dye molecules were determined from the detected blinking events according to an algorithm described in [83]. The so-called “orte-matrix” was produced, which contained information about coordinates and measurement precision for each molecule point. Based on this, “orte-matrix” (“orte”, German for “places/loci”) further evaluation procedures were applied and artificial images of the cell nuclei were constructed.

Mathematical procedures and algorithms, as described in detail in [56,58,81], use these raw coordinate data of the “orte-matrix” instead of processed images. The algorithms applied are based on Ripley’s point-to-point distance frequency measures for structure information [84]. Point-to-point distances were measured by distances from each given central point to its peripheral points. Without regard for absolute position information, the graphical display of the envelope of the absolute or relative distance frequencies can be used for structure interpretation [56,81]. A random point distribution resulting in a homogeneous point-to-point distance distribution leads to a linear increase of the distance frequency curve. Cluster formation corresponding to more frequent smaller distances results in a peak at small distance values. The width of the peak gives the cluster diameter. The area under the peak is a measure relative of the point density in such a cluster. An additional mostly broader peak at higher distance values indicates the distances between the points of two neighboring clusters.

### 2.6. MTT Assay Method

The cells were plated on a 96-well culture plate at concentrations ranging from 3 × 10^3^ to 4 × 10^3^ per well. Volume of medium (DMEM containing 10% fetal calf serum, 1% penicillin/streptomycin) in each well was 100 μL. The cells were cultivated at 37 °C in 5% CO_2_ atmosphere for 24 to 30 h after seeding. Then the nanoparticles were added in two different concentrations (8 or 16 µL/mL; 0.4 or 0.8 μg/mL). For each treatment (IR and/or NPs), seven technical repeats were cultured on a 96-well culture plate. After an 18-h nanoparticle incubation, the nanoparticles were replaced with 100 µL of a fresh cultivation medium and the cells were irradiated as described in Section 2.2.

MTT assay was performed 4 or 72 h post-irradiation according to the manufacturer’s protocol. Ten microliters of the MTT dye (3-(4,5-dimethylthiazol-2-yl)-2,5-diphenyltetrazolium bromide, Duchefa Biochemie, Haarlem, Netherland, cat. No. M1415) were added to each well (the final concentration of MTT 0.5 mg/mL) and the plates were incubated at 37 °C for 3 h. After that, the volume of medium with MTT was discarded and 100 μL DMSO/1N HCl per well were added and thoroughly mixed for 15 min to solubilize the formed formazan crystals. The absorbance of diluted formazan was measured at 570 nm using Infinite 200 PRO microplate reader (Tecan, Männedorf, Switzerland).

### 2.7. NP Cytotoxicity and Radiosensitization Test by Colony Formation (Clonogenic) Assay (CFA)

The cells were plated on 6-well plates at a density of 300 cells per well in 2 mL of the culture medium (DMEM containing 10% fetal calf serum, 1% penicillin/streptomycin) and let settle for 24 h in the incubator (37 °C/5% CO_2_). Subsequently, GNPs (10-nm Aurion, see Section 2.1. for details) at a concentration of 8 µL/mL (0.4 μg/mL) were added. After an 18-h incubation, the GNP-containing medium was replaced with a fresh one without NPs and the cells were irradiated with γ-rays as described in Section 2.2. After the next 9 days, the formed colonies were fixed with ice-cold methanol (1 mL per well for 30 min), stained with 1% trypan blue (1 h), and counted using ImageJ software v1.53m [85,86]. The survival of non-irradiated cells without GNPs was always measured to obtain the plating efficiency (PE). The cell surviving fractions (SF) were counted using PE and the following equations [87]. For each treatment (IR, GNP) condition, the cells were seeded in triplicates per plate, the experiment repeated at least three times, and the results averaged. The displayed values are means ± SD.

$$PE=\frac{number\;of\;colonies\;formed\;\left(average\right)}{number\;of\;cells\;seeded}\times 100\%$$

$$SF=\frac{number\;of\;colonies\;formed\;after\;irradiation\;\left(average\right)}{number\;of\;cells\;seeded\times PE}\times 100\%$$

### 2.8. Real-Time xCELLigence Adhesion/Proliferation Assay

The xCELLigence Real-Time Cell Analysis (RTCA) instrument (ACEA Bio Co, LTD, Hangzhou, China) is a non-invasive, label-free, and real-time cellular impedance-based assay that monitors cell adhesion, which is proportional to cell viability and proliferation, in real time. To gain a more complete view of (cancer) cell killing in dependence on radiation dose and GNP concentration, the xCELLigence instrument was used to capture dynamic cell behavior that can be missed by labor-intensive endpoint methods.

In our experiments 2500, 4000, 5500 and 7000 SkBr3 cells were seeded in the E-plate 16 (ACEA Biosciences, San Diego, CA, USA, cat. No. 00300600890) in technical duplicates. The cell culture medium (DMEM containing 10% fetal calf serum, 1% penicillin/streptomycin) was pipetted to reach the total 200 μL volume in each well. The E-plate 16 was inserted into the RTCA SP Station placed in an incubator (37 °C/5% CO_2_). After the 1–3 h cell adhesion step, the assay started as the RTCA SP Station was switched on. Twenty-four to twenty-seven hours after seeding, the medium was completely removed and replaced with a new one (992 μL) supplemented with 8 μL of 10-nm Aurion GNPs (the final concentration 1.5 × 10^12^ particles/mL, 0.4 μg/mL). After an 18 h-incubation (5% CO_2_/37 °C), the medium with GNPs was removed and replaced again with a fresh one, free of GNPs. Immediately after changing the medium, cells on the E-plates were irradiated as described in Section 2.2. The E-plates were then inserted back into the RTCA SP Station and cultivated for another 100 h.

### 2.9. Cell Cycle Analysis by Flow Cytometry

The Muse Cell Cycle Assay Kit (Merck Millipore, Burlington, MA, USA, cat. No. MCH100106) and Muse Cell Analyzer (Merck Millipore, Burlington, WA, USA) were used to determine changes in SkBr3 cell cycle distribution upon irradiation (1 + 1 Gy, 2 Gy or 4 Gy) in the presence (18-h incubation) or absence of Aurion gold nanoparticles (0.4 μg/mL). The procedure was performed as described in the manufacturer’s protocol (available online at https://www.luminexcorp.com/muse-cell-cycle-kit/#documentation; accessed on 25 November 2021) or in [55]. SkBr3 cells were seeded on 6-well plates at a density of 2 × 10^5^ cells per well in 2 mL of the culture medium (DMEM containing 10% fetal calf serum, 1% penicillin/streptomycin). The cells were irradiated with 0, 1 + 1, 2 or 4 Gy γ-rays (see Section 2.2) and assessed 30 min and 4 h post-irradiation. Guava InCyte software, ver. 3.1.1. (Millipore, Burlington, MA, USA) was used for the data analysis, with the same gates in all samples.

### 2.10. Western Blotting

The cells were harvested at the defined time points after irradiation (4 Gy) and lysed (5 min) on ice in RIPA buffer (Thermofisher Scientific, Rockford, IL, USA, cat. No. 89900), supplemented with protease and phosphatase inhibitors (Thermofisher Scientific, cat. No. 78442). The lysates were sonicated in three 10 s intervals (Hielscher UP100H Lab Homogenizer, Hielscher Ultrasonics, Teltow, Germany) and clarified by centrifugation at 15,000× *g* rpm for 10 min. The supernatants were collected and stored at −80 °C. The protein concentrations of samples were determined by using Pierce™ BCA Protein Assay Kit (Thermofisher Scientific, cat. No. 23227). γH2AX (histone H2AX phosphorylated at ser139) was quantified on standard 12% SDS-PAGE gels while protein 53BP1 on precasted SDS-PAGE gels (NuPAGE 3–8% Tris Acetate, Thermofisher Scientific, cat. No.: EA0375BOX). Proteins were transferred onto the PVDF membrane (Thermofisher Scientific, cat. No.: 88518) and the membrane with target proteins were blocked in 5% non-fat milk in TBS-T (20X TBS Buffer, Thermofisher Scientific, cat. No. 28358) for 90 min at room temperature. The incubation of membranes with the primary antibodies was performed overnight at 4 °C. The primary antibodies, which were tested for the specificity: anti-phospho histone H2AX (Ser139) antibody, clone JBW301 (Merck-Millipore, Burlington, WA, USA cat. No.: 05-636-I, 1:1000) and anti-53BP1 antibody (Cell Signaling, Danvers, MA, USA, cat. No. 4937, 1:250). Finally, the membrane was incubated with the secondary HRP (horse radish peroxidase) conjugated antibodies (Thermofisher Scientific, cat. No. 31430 and cat. No. 31460, 1:2000) for 1 h and afterward, chemiluminescence was detected by scanner Amersham^TM^ Imager 680 (GE, Healthcare, Bio-Science AB, Uppsala, Sweden).

## 3. Results

### 3.1. Extent, Repair Kinetics and Persistence of Radiation-Induced DNA Double Strand Breaks (DSBs) in the Nuclear DNA of SkBr3 Mammary Carcinoma Cells Incubated with 10-nm GNPs

SkBr3 mammary carcinoma cells were selected as a model to study the nanoparticle-mediated radiosensitization because most mammary tumors are treated by irradiation. Moreover, radiotherapy of mammary tumors is often difficult for their potentially close proximity to the heart. Thus, the possibility to reduce the radiation dose to protect the heart (or other organs) with the preserved therapeutic effect on the tumor by the means of nanoparticle-mediated radiosensitization would be a significant improvement.

The cells were cultured in 6-well plates and incubated or not incubated with 0.4 μg/mL (for MTT also 0.8 μg/mL) GNPs for 18 h (at 37 °C). Subsequently, both the nanoparticle-treated (GNP(+)) and untreated (GNP(−)) cells were irradiated with different doses of γ-rays, specifically 2 or 4 Gy, where the 2 Gy dose was delivered either as a single exposure or two 1 Gy doses separated by 30 min (further referred to as 1 + 1 Gy dose). The doses were selected for their clinical relevance in cancer radiotherapy; moreover, the DNA damage after 4 Gy irradiation is supposed to be preferentially repaired by other mechanisms compared to doses up to 2 Gy [88]. The following cell specimens were obtained: (a) non-irradiated controls without nanoparticles to quantify the DSB background in SkBr3 cells; (b) non-irradiated cells incubated with the indicated concentration of GNPs to measure the inherent nanoparticle cytotoxicity; (c) irradiated cells without GNPs to describe the natural response of SkBr3 cells to increasing radiation doses; and (d) irradiated cells treated with GNPs and exposed to the same radiation doses as in (c) to study combined effects of different nanoparticle concentrations and radiation doses on the nuclear DNA damage, cell cycle and cell viability.

First, we explored at the microscale level the hypothesis that irradiated GNPs enhance the extent of DSBs generated in the nuclear DNA by ionizing radiation (IR), as it could be predicted based on physical interaction between IR and heavy metal (high-*Z*) atoms. In addition, we analyzed whether GNPs increase the complexity of radiation-induced DSBs and slow down the kinetics of their repair during a long time window after irradiation (0.5 to 24 h PI). Both the enhanced DSB induction and DSB complexity due to GNPs may lead to a higher occurrence of late DSBs. Hence, we also compared the persistence of non-reparable DSBs at the latest PI time (24 h PI) for cells irradiated in the presence or absence of GNPs.

**Figure 1:**
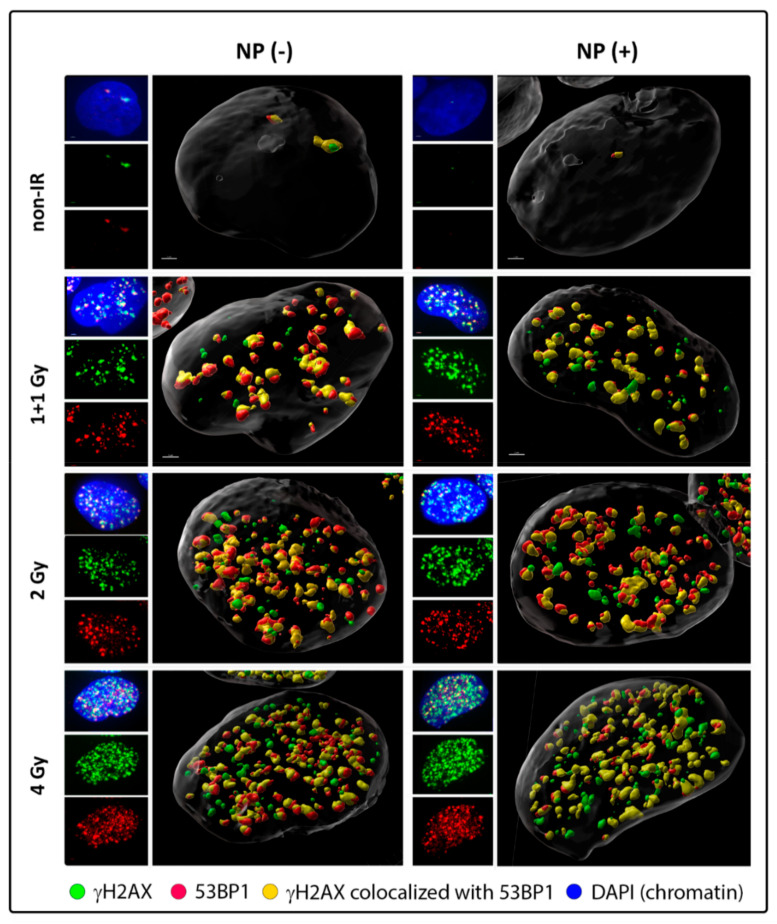
The quantity, morphology and extent of mutual colocalization of γH2AX (**green**) and 53BP1 (**red**) repair foci generated within the nuclear DNA of SkBr3 mammary carcinoma cells with different doses of γ-rays in absence (−) or presence (+) of GNPs (0.4 μg/mL for 18 h at 37 °C prior to irradiation). The figure shows the situation at 0.5 h PI, i.e., the post-irradiation time of repair focus maximal occurrence. The micro-morphology and colocalization of foci in 3D space were reconstituted by Imaris software. For better visualization, γH2AX foci colocalizing with 53BP1 are displayed in yellow while all 53BP1 foci (including those colocalizing with γH2AX) in red.

**Figure 2:**
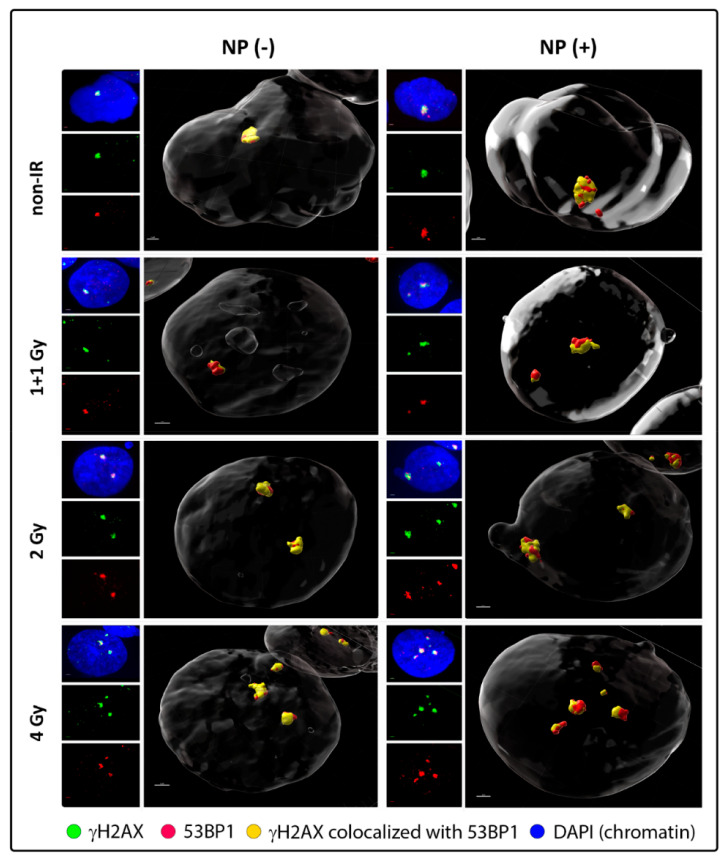
Persistence of non-reparable radiation-induced DSBs in SkBR3 cells as measured by γH2AX (**green**) plus 53BP1 (**red**) repair foci persisting in cell nuclei 24 h PI. Repair focus numbers, their micro-morphology visualized in 3D space by Imaris software, and γH2AX + 53BP1 colocalization are cross-compared for different γ-ray doses (1 + 1, 2 and 4 Gy) and cells irradiated in the absence (**left column**) or presence (**right column**) of GNPs (0.4 μg/mL for 18 h at 37 °C prior to irradiation). For better visualization, γH2AX foci colocalizing with 53BP1 are displayed in yellow while all 53BP1 foci (including those colocalizing with γH2AX) in red.

To this point, we visualized DSBs by labeling of γH2AX and 53BP1 repair foci and monitored their formation and disassembly (DSB repair) by using a high-resolution immunofluorescence confocal microscopy of spatially conserved (3D), fixed cells (Figure 1 and Figure 2; Supplementary Materials, Figure S1). γH2AX foci have been recognized as the specific marker of DSBs that can be detected in cell nuclei in the early minutes of PI. However, especially in cancer cells, γH2AX foci may be imprecisely developed (e.g., diffuse) and accompanied by a relatively strong background that can be difficult to distinguish from the real foci. Hence, in order to increase the precision of DSB scoring [89,90], we also labeled 53BP1 repair protein, which is involved in both DSB repair pathways in human cells—non-homologous end-joining (NHEJ) and homologous recombination (HR)—and extensively colocalize with (theoretically all) γH2AX foci. Moreover, 53BP1 foci provide higher precision to estimate DSB complexity at the microscale [41].

Western blotting confirmed the specificity of both anti-γH2AX and anti-53BP1 antibodies used (Supplementary Materials, Figure S2). γH2AX and 53BP1 foci in specially fixed nuclei of SkBr3 cells were separately detected and visually reconstituted in 3D by Imaris software (Oxford Instruments, Oxford, UK). Visual comparison of randomly selected GNP(−) and GNP(+) cells prior to irradiation and in different periods after exposure to 1 + 1, 2 or 4 Gy IR doses did not reveal striking differences in numbers of both generated (Figure 1) or persistent (Figure 2) γH2AX and 53BP1 foci. Batch image processing and thus automatic comparison of focus parameters by Imaris has been found to be extremely problematic for a strong dependence of the analysis on the initial signal (R-G-B) intensity settings, as this intensity differed between samples and went to imprecise focus segmentation.

Hence, we analyzed the numbers, micro-morphology, mutual colocalization, and other focus parameters of γH2AX and 53BP1 foci (e.g., intensity) with a high precision using our newly-developed software (DeepFoci) [70] based on artificial neural networks and deep machine learning. After appropriate training for γH2AX and 53BP1 focus detection, the software provided focus numbers closely comparable to manual analysis by an experienced expert, with the advantage of precisely extracting focus parameters irrespective of the sample intensity or other complicating factors (e.g., background) [70].

**Figure 3:**
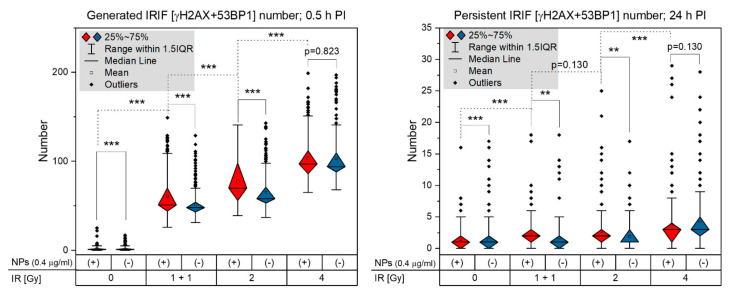
Numbers of generated (**left** graph) and persistent DSBs (**right** graph) as a function of radiation dose and presence of GNPs. DSBs were quantified as colocalized [γH2AX+53BP1] repair foci. The average numbers of [γH2AX+53BP1] repair foci per nucleus are shown together with 1.5 IQR (interquartile range) for SkBr3 breast cancer cells exposed to 1 + 1, 2 or 4 Gy γ-ray doses in the presence (+) or absence (−) of 10 nm GNP (0.4 μg/mL). The cells were fixed at 0.5 h (left graph) or 24 h PI (right graph). The shorter time corresponds to the post-irradiation period with the maximal occurrence of repair foci while the longer time corresponds to the post-irradiation period when the vast majority of DSBs is repaired. Rhombuses contain 50% of dataset values, which lay between the top (75%) and bottom (25%) quartiles. Asterisks denote statistical significance of differences between indicated samples at *p* = 0.05 (*), *p* = 0.01 (**) or *p* = 0.001 (***). Two-tailed *t*-test, two-tailed *t*-test with Welsh correction or Mann-Whitney Rank Sum Test was used depending on the distribution normality of the analyzed variables.

The detailed software analysis of large cell datasets (100–200 cells/cell specimen) revealed significantly higher numbers of colocalized γH2AX and 53BP1 foci (further referred to as [γH2AX + 53BP1] foci) in cells irradiated in presence of GNPs (Figure 3, left graph). The differences in [γH2AX + 53BP1] focus numbers were significant for all studied radiation doses, except for the highest dose of 4 Gy. Nevertheless, the slightly higher mean focus number and variation (1.5IQR intervals, Figure 3, left graph) in GNP(+) cells also point to a mild ‘radiosensitization’ with this highest IR dose; hence, the damage induced by radiation per se has probably already been so high that an additional contribution from GNP-mediated ‘radiosensitization’ is only insignificant. On the other hand, a clearly significant difference between [γH2AX + 53BP1] focus numbers in GNP-treated and non-treated cells was observed also in non-irradiated cells. This result might suggest that GNPs may provoke DNA damaging or other processes leading to enhanced focus numbers even by themselves. Nevertheless, the difference might also reflect very low and highly variable numbers of repair foci in non-irradiated cells and a random selection of cells for the analysis from non-synchronized cell populations.

Similar trends as for the DSB induction (i.e., γH2AX+53BP1 focus evaluation at 30 min PI) were recognized also for the persistence of unrepaired/non-reparable DSBs at 24 h PI (Figure 3, right graph). Again, significantly higher numbers of [γH2AX + 53BP1] foci persisted in cells irradiated in presence of GNPs, which was observed for all radiation doses, except for the highest one (4 Gy). However, in all cases, the significance was lower compared to 30 min PI, suggesting that the higher DSB damage generated by GNPs is mostly buffered by the repair system of SkBr3 cells. Despite this fact, slightly increased presence of unrepaired DSBs is also compatible with occasional generation of more complex DSBs by free radical clouds produced by GNP-emitted secondary (delta) electrons. As only delta electrons with the highest energies can reach the nucleus from the cytoplasm, where GNPs are located [6,46], the proportional increase of complex DSBs could be expected to be low.

**Figure 4:**
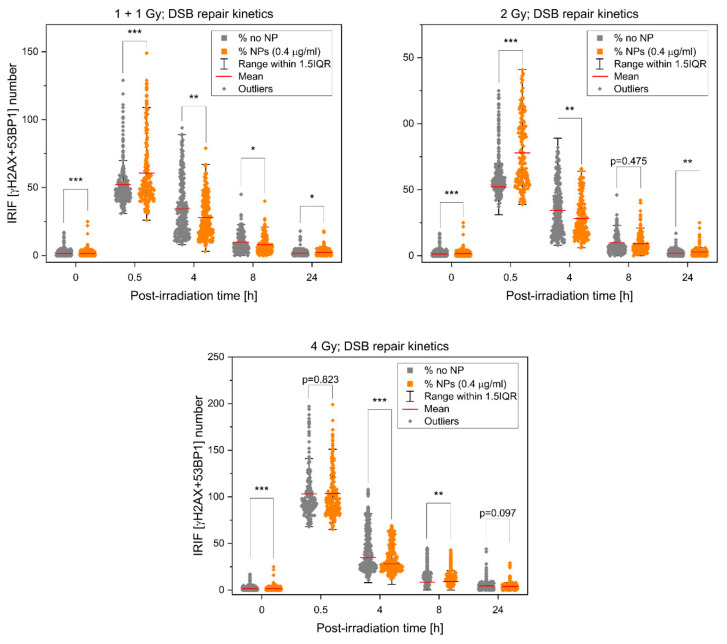
DSB repair kinetics compared for SkBr3 cells exposed to 1 + 1, 2 or 4 Gy of γ-rays in the presence (**orange**) or absence (**gray**) of 10 nm GNPs (0.4 μg/mL). The average numbers of [γH2AX + 53BP1] repair foci per nucleus are shown together with 1.5IQR. For each dose, the cells were fixed at 0.5, 4, 8 and 24 h PI, respectively. The shortest time corresponds to the post-irradiation period with the maximal occurrence of repair foci; at 4 h PI, most of fast (NHEJ) repaired DSBs is already accomplished; at 8 h PI, slow HR is also mostly finished; at the longest PI time (24 h), all DSBs except of those repaired only with difficulty are re-joined. Asterisks denote statistical significance of differences between indicated samples at *p* = 0.05 (*), *p* = 0.01 (**) or *p* = 0.001 (***). Two-tailed *t*-test, two-tailed *t*-test with Welsh correction or Mann-Whitney Rank Sum Test was used depending on the distribution normality of the analyzed variables.

A more detailed view on the radiation response of SkBr3 breast cancer cells treated or not treated with GNPs is provided in Figure 4, which shows DSB repair kinetics for radiation doses of 1 + 1, 2 and 4 Gy and the post-irradiation time window from 0.5 to 24 h PI. For each dose, the cells were fixed at 0.5, 4, 8 and 24 h PI, respectively. The shortest time corresponds to the post-irradiation period with the maximal occurrence of repair foci. Four and 8 h PI time periods were selected to approximate the efficiency of the two main DSB repair pathways, non-homologous end-joining (NHEJ) and homologous recombination (HR), respectively. At 4 h PI, most of fast (NHEJ) repaired DSBs is already accomplished; at 8 h PI, slow HR is also mostly finished. At the longest time PI (24 h), all DSBs but those repaired only with difficulty are re-joined. With the exception of already described higher γH2AX and 53BP1 focus numbers on GNP(+) cells at 0.5 h PI, especially those irradiated with 1 + 1 and 2 Gy doses, it can be seen that DSB repair kinetics is not compromised by GNPs. However, slightly but statistically significantly higher numbers of persistent [γH2AX + 53BP1] foci in cells irradiated with GNPs point to occasional generation of more complex DSBs due to GNP presence.

### 3.2. Colocalization of γH2AX with 53BP1 and Morphology of DSB Repair Foci at the Microscale

**Figure 5:**
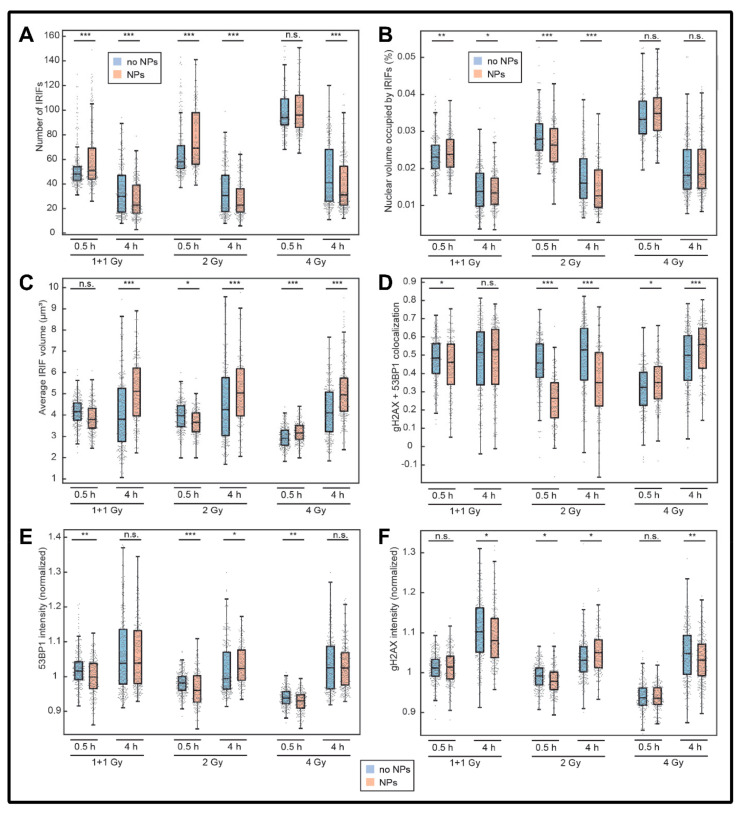
Morphology of DSB [γH2AX+53BP1] repair foci compared at the microscale for SkBr3 cells exposed to 1 + 1, 2 or 4 Gy of γ-rays in the presence (**orange**) or absence (**blue**) of GNPs (0.4 μg/mL). (**A**) Average [γH2AX+53BP1] repair focus numbers per nucleus at 0.5 and 4 h PI times, when the focus morphology was analyzed (see text related to Figure 3 and Figure 4 for more information on DSB numbers). (**B**) Nuclear volumes (%) occupied by [γH2AX+53BP1] repair foci. (**C**) Average [γH2AX+53BP1] repair focus volumes [μm^3^]. (**D**) The extent of γH2AX + 53BP1 signal colocalization. (**E**) Intensity of 53BP1 foci normalized for the background signal intensity. (**F**) Intensity of γH2AX foci normalized for the background signal intensity. Asterisks denote statistical significance of differences between indicated samples at *p* = 0.05 (*), *p* = 0.01 (**) or *p* = 0.001 (***); ns = not significant. Two-tailed *t*-test, two-tailed *t*-test with Welsh correction or Mann–Whitney Rank Sum Test was used depending on the distribution normality of the analyzed variables.

To evaluate radiation-mediated DSB induction and repair as a function of GNP pre-treatment in more detail, the colocalization of γH2AX foci with 53BP1 repair protein was analyzed together with numerous morphological parameters of γH2AX and 53BP1 repair foci at the microscale. As demonstrated by Figure 5B, the nuclear volume [%] occupied by colocalized [γH2AX + 53BP1] repair foci at 0.5 h PI increased linearly with the dose in all samples, in agreement with the growing numbers of these foci (Figure 5A). In contrast, this increase was almost absent in the later PI time (4 h PI), suggesting that the repair kinetics increased with the dose for the given dose range studied (up to 4 Gy). Even the exposure to the highest 4 Gy dose does not lead to the exhaustion of DSB repair capacity of SkBr3 cells.

**Figure 6:**
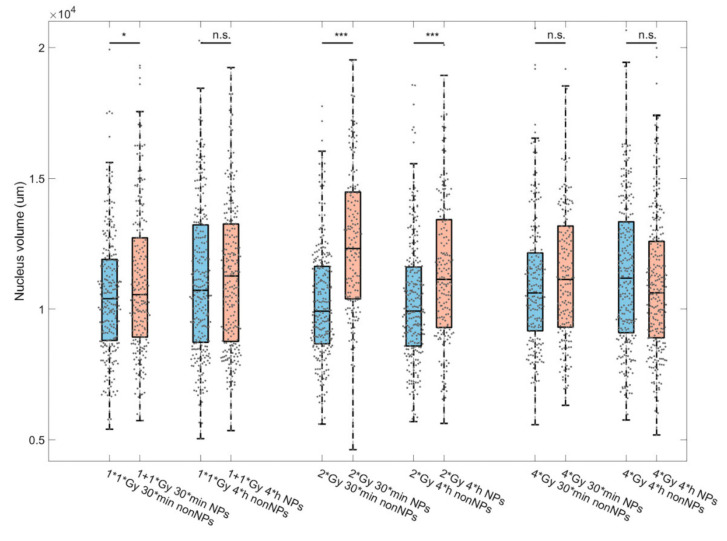
Volumes [μm^3^] of SkBr3 cell nuclei compared for cell specimens exposed to 0, 1 + 1, 2 or 4 Gy of γ-rays in the presence (**orange**) or absence (**blue**) of GNPs (0.4 μg/mL). Asterisks denote statistical significance of differences between indicated samples at *p* = 0.05 (*), *p* = 0.01 (**) or *p* = 0.001 (***); ns = not significant. Two-tailed *t*-test, two-tailed *t*-test with Welsh correction or Mann–Whitney Rank Sum Test was used depending on the distribution normality of the analyzed variables.

When the cells irradiated in the presence or absence of GNP are compared, the occupied volumes were (in most cases) slightly lower in GNP-treated cells. As more [γH2AX + 53BP1] repair foci are detected in GNP(+) cells at 0.5 h PI and these foci are on average slightly bigger at 4 h PI compared to GNP(−) cells, this unexpected result could probably be explained by slightly bigger volumes of GNP(+) cell nuclei, as suggested by Figure 6.

Interestingly, the volumes [μm^3^] of [γH2AX + 53BP1] repair foci were smaller at 0.5 h PI but larger at 4 h PI in GNP-treated cells relative to the untreated controls (Figure 5C). This might be explained by higher numbers of smaller [γH2AX + 53BP1] repair foci in GNP(+) cells at 0.5 h PI and, on the other hand, a higher persistence of more complex DSBs and therefore larger repair foci at 4 h PI. The exception from this trend were the cells exposed to 4 Gy and evaluated at 0.5 h PI, where [γH2AX + 53BP1] focus volumes in GNP(+) cells were slightly larger than in GNP(−) cells. However, the focus numbers in both these highly irradiated GNP(−) and GNP(+) cell specimens are high and the focus volumes therefore small. Even faint differences in sample staining/intensity might therefore influence the analysis. It should be noted that [γH2AX + 53BP1] focus volumes significantly decreased for the 4 Gy samples at 0.5 h PI but not 4 h PI. This suggests that the formation of [γH2AX + 53BP1] foci is comparably fast for 1 + 1 and 2 Gy samples but significantly decreased for the 4 Gy samples. The 4 Gy dose thus does not exhaust DSB repair system of SkBr3 cells in a longer-time perspective but saturates its capacity at the initial phase of repair (Figure 5C).

At 0.5 h but not 4 h PI, the colocalization between γH2AX and 53BP1 repair foci was lower for the both GNP(+) and GNP(−) cells at the 4 Gy dose compared to the 2 Gy and 1 + 1 Gy doses (Figure 5D). Together with micro-morphological analysis already described in previous paragraphs, this observation points to temporary exhaustion of DSB repair in its initial phase after application of high (≥4 Gy) radiation doses. However, this initial “saturation” of DSB repair did not decrease the repair kinetics and overall repair capacity, as could be deduced from numbers of γH2AX/53BP1 foci persisting in cells at 4 h PI (Figure 5A).

In addition, at both post-irradiation periods studied (0.5 and 4 h), the colocalization of γH2AX and 53BP1 repair foci was for all doses but the 4 Gy dose lower in GNP-treated cells (for the 1 + 1 Gy dose at 4 h PI the difference between GNP(−) and GNP(+) cells was statistically insignificant). This result might suggest that GNPs, for still unknown reasons, slightly reduce the amount of “active” 53BP1 protein available to cells or its binding to γH2AX foci. Similar to repair system saturation described above for high radiation doses, the reduction of 53BP1 colocalization with γH2AX foci due to presence of GNPs (Figure 5D) was not functionally manifested by a decrease of DSB repair kinetics or capacity (Figure 5A).

In accordance with the lower colocalization of γH2AX and 53BP1 repair foci in GNP-treated cells at 0.5 h PI, intensity of the normalized 53BP1 focus signals was also lower in these cells (Figure 5E). The exception from this correlation was the pair of GNP(−)/GNP(+) samples exposed to 4 Gy, where presence of GNPs reduced the 53BP1 focus intensity (Figure 5E) but not the colocalization of γH2AX foci with 53BP1 (Figure 5D). Interestingly, at this highest radiation dose studied, significantly lower focus colocalization and 53BP1 intensity compared to the lower doses was observed not only for GNP(+) but also GNP(−) specimens. This suggests that the 4 Gy dose itself exhausts 53BP1 protein, leaving a much smaller effect of GNP(+) difficult to measure with a given precision of confocal microscopy. The intensity comparison for γH2AX showed the same trend but in a much less significant extent (Figure 5F). The phosphorylation of histones H2AX at ser139 thus does not seem to be influenced by the presence of GNP in irradiated SkBr3 cells.

**Figure 7:**
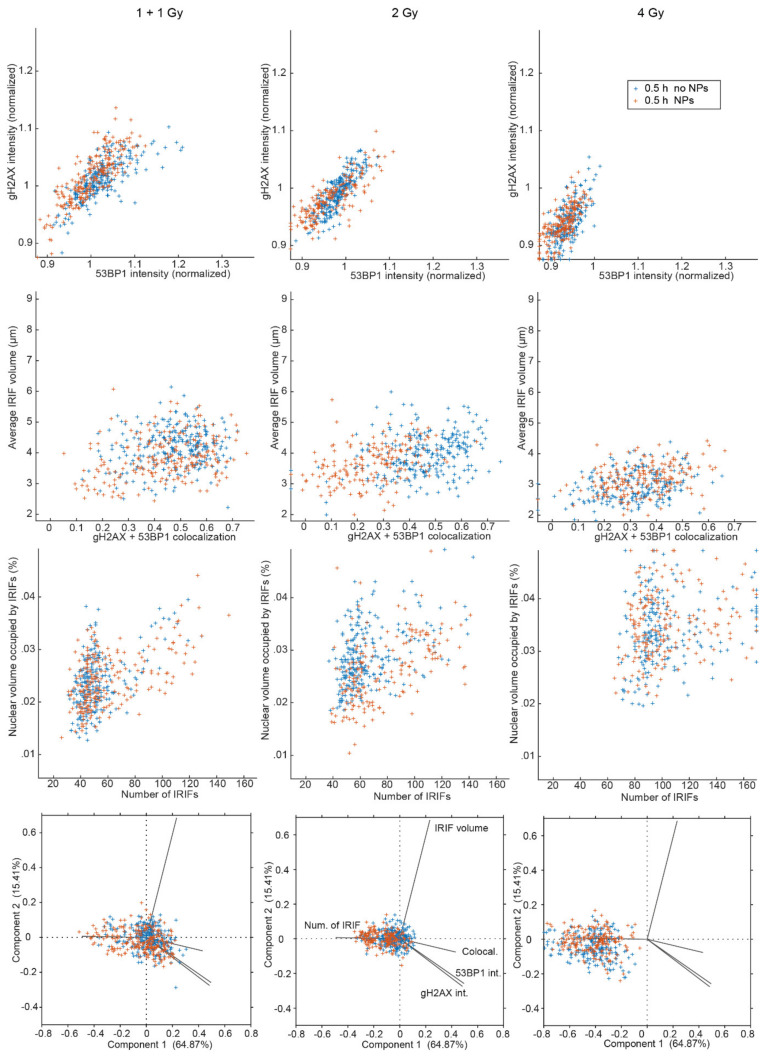
Correlations and principal component analysis (PCA) of multiple γH2AX and 53BP1 repair focus parameters compared for GNP(−) (**blue**) and GNP(+) (0.4 μg/mL, **orange**) SkBr3 cells irradiated with indicated doses of γ-rays and analyzed at 0.5 h PI. See the main text for details.

**Figure 8:**
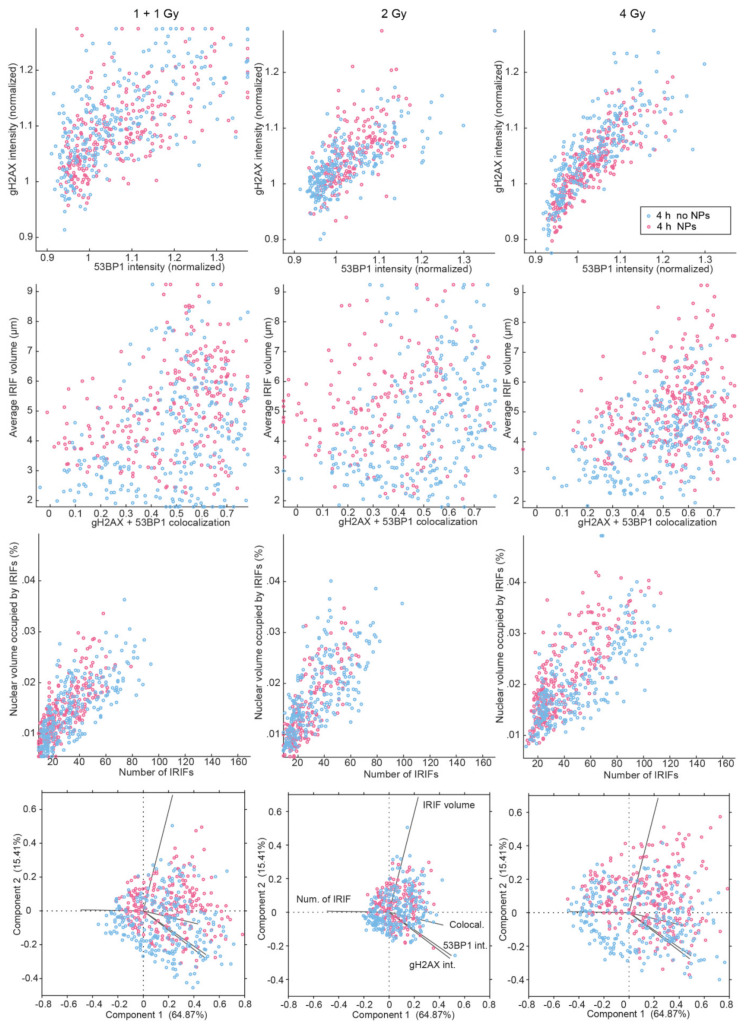
Correlations and principal component analysis (PCA) of multiple γH2AX and 53BP1 repair focus parameters compared for GNP(−) (**blue**) and GNP(+) (0.4 μg/mL, **red**) SkBr3 cells irradiated with indicated doses of γ-rays and analyzed at 4 h PI. See the main text for details.

Correlations between selected parameters and results of principal component analysis (PCA) for γH2AX and 53BP1 repair foci in GNP(−) and GNP(+) cells exposed to 1 + 1, 2 or 4 Gy γ-rays are shown for 0.5 h (Figure 7) and 4 h PI (Figure 8). For 0.5 h PI, a linear correlation was observed for the normalized γH2AX and 53BP1 signal intensities (Figure 7, the first line). Plotting the average [γH2AX + 53BP1] focus volume against the extent of colocalization between γH2AX and 53BP1 foci revealed a marked (2 Gy) or less prominent (1 + 1 Gy) shift between GNP(−) and GNP(+) populations with similar focus volumes for both populations but different extent of γH2AX and 53BP1 focus colocalization. The population with the lower colocalization mostly included GNP(+) cells, with the exception of the 4 Gy specimen where the two subpopulations cannot be distinguished (Figure 7, the second line). When the average volume of the nucleus occupied by [γH2AX + 53BP1] foci is displayed against the number of [γH2AX and 53BP1] foci, two cell subpopulations with approximately twice the difference in the focus numbers, but a smaller difference in nuclear volumes occupied by foci, can be distinguished, both within GNP(+) and GNP(−) cell specimens. The subpopulation with higher focus numbers and nucleus volumes occupied by foci is then markedly more prominent for GNP(+) cells, again except the 4 Gy specimen (Figure 7, the third line). Together with larger nucleus volumes (Figure 6) of GNP(+) cells, these results point to an increased proportion of G2-phase cells in GNP(+) specimens compared to GNP(**−**) ones.

Principal component analysis (Figure 7, the last line) disclosed a correlation between the intensities of γH2AX and 53BP1 foci, and, to some extent, a correlation of these parameters with the extent of γH2AX and 53BP1 foci. On the other hand, all these parameters anti-correlated with the number of foci per nucleus. The [γH2AX + 53BP1] focus volume was then independent of the remaining parameters studied. With a significant overlap though, GNP(−) and GNP(+) cells can partially be separated by PCA of DSB repair focus parameters. The contribution of individual parameters to component 1 and component 2 and the discrimination strength of the two components is also shown.

Results of multiparameter repair focus analysis for 4 h PI were similar to those for 0.5 h PI with some important differences. First, a higher dispersion of parameters suggested a higher heterogeneity of foci at this later PI time (Figure 8). This could be explained by the presence of foci in different phases of repair and/or foci repaired by different mechanisms [40]. As the foci are more developed at 4 h compared to 0.5 h PI, their micro-morphological variability due to different repair mechanisms could be more prominent.

Second, two subpopulations of cells differing from each other by about twice the number of [γH2AX + 53BP1] foci, observable at 0.5 h PI within cell population of both GNP(−) and GNP(+) specimens (at 1 + 1 and 2 Gy doses; Figure 7, third line), are no longer distinguishable at 4 h PI (Figure 8, third line). However, lower [γH2AX + 53BP1] focus numbers compared to GNP(−) cells are now (4 h PI) evident in GNP(+) cells, especially at the 4 Gy dose. This is the opposite situation compared to 0.5 h PI, when GNP(+) cells contained more foci (Figure 7, third line). Moreover, the nature of this focus number shift is different— it is less prominent than at 0.5 h PI but, on the other hand, occurs in more cells. Finally, GNP(+) cells had larger nuclear volumes than GNP(−) cells in all samples at both 0.5 and 4 h post-irradiation times. The exception occurred for the 4 Gy dose, where this trend was reversed (see also Figure 6). This suggests that the highest radiation dose used in combination with GNPs may predominantly kill larger (G2) cells, which is compatible with the above described results (e.g., absence of the increase of the cell subpopulation with high [γH2AX + 53BP1] focus numbers in GNP(+) cells exposed to 4 Gy).

### 3.3. SMLM Analysis of γH2AX Focus and H3K9me3 Heterochromatin Domain Topology at the Nanoscale

**Figure 9:**
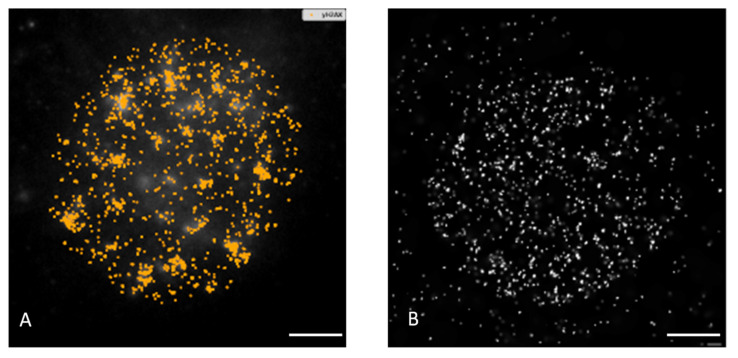
Typical examples of artificial images obtained from data of the “orte matrix”. (**A**) γH2AX signals (**orange**) 30 min after incubation of SkBr3 cells with GNPs and 2 Gy radiation exposure. This image is merged with the real widefield DAPI image (**shadowed**). (**B**) H3K9me3 heterochromatin signals without DAPI image of the cell nucleus of a non-irradiated control with GNPs. Scale bar 2 µm.

In the next step, we have analyzed DSB repair foci at the molecular (nanoscale) level. In addition, chromatin architecture and its potential changes after GNP incubation, irradiation and DSB repair were followed using SMLM with the resolution up to 10 nm. Heterochromatin domains labeled with the anti-H3K9me3 antibody, which are known to undergo extensive reorganizations upon irradiation, were selected as an object of interest. For the visualization of typical results, artificial images of the labeled γH2AX and H3K9me3 molecules were prepared from the “orte matrices” of the obtained signal coordinated in the respective color plane. For better segmentation, these images could also be merged with the real widefield DAPI image of a cell nucleus. Examples are presented in Figure 9.

For each experimental condition, more than 20 cell nuclei were analyzed (note that thousands of images in each color channel are necessary for SMLM of a single nucleus). The GNPs were distributed in the cytosol and formed clusters (for comparison see [14]). A considerable part of the GNPs was found close to the cell membrane, which could be verified by separate experiments detecting particle induced quenching of fluorescence (data not shown).

**Figure 10:**
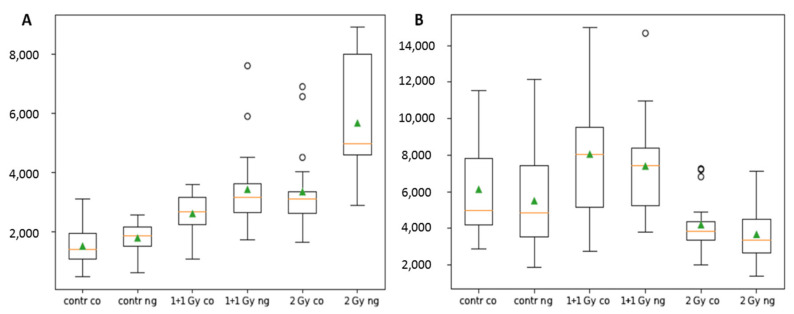
Absolute number of fluorescent points detected per image (z-section of 500 nm) for the different experiment; contr = non-irradiated control; 1 + 1 Gy = radiation scheme 1 G—30 min—1 Gy; ng = GNPs (nano-gold); co = control without GNPs. Each detected point represents a fluorescent antibody against the γ-H2AX molecule (**A**) or the H3K9 methylation site (**B**) as detected in an image plane of the cell nucleus. Blinking events within the localization precision of the specific image were registered as one point. The boxplots show the mean point number of the detected nuclei (**green triangle**), the median (**orange line**), the lower and upper quantile (box), and the value range within ± 2 standard deviations (**black line**). The black circles refer to values that are differing more than 1 box length from the median.

For all cell nuclei, the number of γH2AX tags was determined 30 min post (last) irradiation (Figure 10A). Incorporation of GNPs did not significantly increase the number of recorded tags in the non-irradiated control. For the irradiated cells without GNPs, the number of tags increased as compared to the non-irradiated controls but the irradiation schemes of 1 + 1 Gy or 2 Gy did not show any effect. For these samples, adding of GNPs led to an increase of γH2AX tags, which was highly significant for 2 Gy. In addition, the variability of the tag numbers was much higher for cells under this condition indicating a heterogeneous damage pattern for different cells, which may reflect a variable GNP incorporation. These different results for 1 + 1 Gy and 2 Gy irradiation schemes indicate that the radiation time-out of 30 min between the two radiation fractions may induce an up-regulated repair process.

Signal counting of heterochromatin-associated H3K9me3 tags 30 min post (last) irradiation (Figure 10B) reflected a high variability of the cells, which may be due to low numbers of cells analyzed (given by technical limits of the method) and a random bias in selection of cells in different cell cycle phases. Therefore, no statistically significant differences were observed for the different experiments. However, the tag counts for the single 2 Gy dose were slightly lower and less variable compared to the same dose but delivered as two 1 Gy doses separated by 30 min (1 + 1 Gy sample). This may indicate that more cells are retained in the G2/M cell cycle arrest after the higher single dose (G2/M cells have more condensed chromatin [91]). Interestingly, with the exception of cells exposed to the 1 + 1 Gy dose, the H3K9me3 tag counts were more heterogeneous for GNP(+) compared to GNP(−) cells, pointing to more open configuration of (some) heterochromatin domains in the former cells. All these observations are in a good accordance with the results obtained at the microscale. However, in general, the incorporation of GNPs did not show a dramatic effect on heterochromatin tag numbers as compared to the cells equally treated but without GNPs.

**Figure 11:**
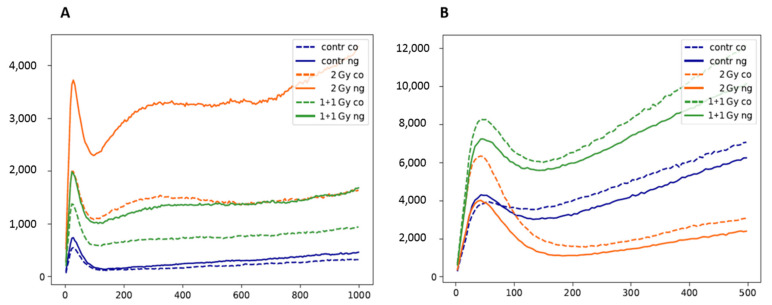
Ripley’s distance frequency analysis at 0.5 h post-irradiation. The absolute frequencies of pairwise distances (**ordinate**) vs. the distances in nm (**abscissa**) are presented for the different types of experiments; contr = non-irradiated control; 1 + 1 Gy = radiation scheme 1 Gy—30 min—1 Gy; ng = GNPs (nano-gold); co = control without GNPs. (**A**) Pairwise distance numbers of γ-H2AX labeling signals. (**B**) Pairwise distance numbers of H3K9me3 labeling signals.

In addition to molecule signal counting, the pairwise distances between the labeling points were determined and counted. In Figure 11, the results of the Ripley’s distance frequency curves are presented. All curves show a peak for γH2AX clustering in the distance range below 150 nm (Figure 11A). The non-irradiated control samples showed a low peak indicating only a few background γH2AX clusters. In this case, the GNPs did not show any effect in contrast to the irradiated samples. Although the size of the clusters (=width of the cluster peak) did not change significantly, the absolute number of clusters increased for the samples with GNP incorporation. This increase of clusters was associated with an increase of the cluster density, which was reflected by the second broader peak at about 300 nm distances. This second peak was always more pronounced in the irradiated samples with GNPs indicating an increased damage density in a smaller part of the cell nuclei as compared to the samples without GNPs. Comparing the 1 + 1 and 2 Gy-irradiation schemes, it is obvious that the 30 min irradiation time-out was used by the cells for fast repair processes of DSBs.

This increased repair activity could also be responsible for the results of Ripley’s distance frequency curves for H3K9me3 tag distances (Figure 11B). Here, the data showed a broad peak and continuous increase of the curves especially for the 1 + 1 Gy samples. This indicates a strong relaxation of heterochromatin towards a random distribution of labeling points, which means that the clusters of heterochromatin undergo opening to improve the accessibility for repair proteins. In the samples exposed to a single 2 Gy dose, the compaction of heterochromatin was considerably stronger, even stronger than in the non-irradiated samples. However, while the number of strongly condensed clusters was increased, the larger distance frequencies indicated a nearly random distribution. This correlates to a more relaxed state outside the clusters and with a more open accessibility to the heterochromatin. In all cases, the incorporation of GNPs influenced the spatial organization and architecture of heterochromatin.

### 3.4. Cell Cycle Analysis

**Figure 12:**
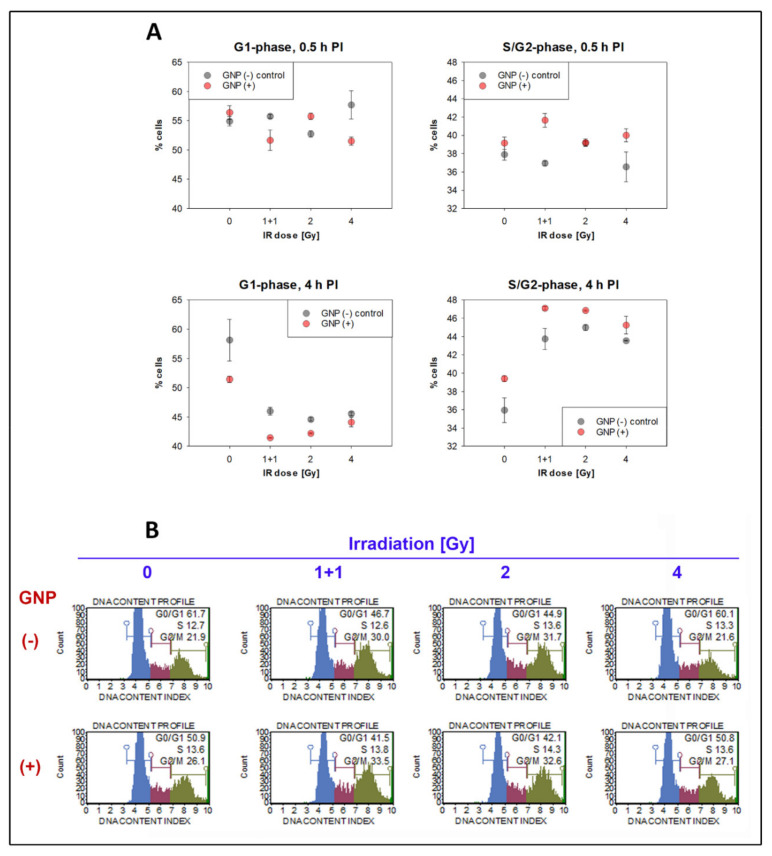
Flow cytometry analysis of cell cycle distribution of SkBr3 cells in dependence of the irradiation dose and GNP incubation (0.4 μg/mL for 18 h). (**A**) The mean values (±SE) of G1 vs. S/G2/M cells for all treatments and their combinations. Controls without GNP: gray symbols, GNP-treated cells: red symbols (**B**) Examples of raw flow cytometry data for non-irradiated control SkBr3 cell specimens incubated (+) or not incubated (−) with GNPs and these specimens examined 4 h after exposure to the indicated doses of γ-rays. The conditions of GNP incubation were the same as in A. Cell populations in specific phases of the cell cycle are labeled as follows: G0/G1—blue, S—purple, G2/M—green.

Microscale and nanoscale analyses of repair foci and nuclear chromatin described in previous sections revealed the existence of an increased subpopulation of cells with larger nuclei and approximately twice the number of γH2AX and 53BP1 repair foci in GNP(+) cell specimens. This finding strongly points to possible changes of the cell cycle distribution due to the presence of GNPs. To test this hypothesis, flow cytometry measurements of the cell cycle were performed for all samples at 0.5 and 4 h PI (Figure 12A). For all radiation doses (1 + 1, 2 and 4 Gy) a significant decrease of G1 cells accompanied by a corresponding increase of S/G2/M cells was observed at 4 h but not 0.5 h PI.

This decrease of G1 cells and increase of S/G2/M cells was only slightly but systematically more prominent for the cells irradiated in presence of GNPs and assessed at 4 h PI. Interestingly, some signs of this trend were observed already at 0.5 h PI and in non-irradiated cells. It should be noted that though S, G2 and M phase cells were evaluated as one cell category in Figure 12, the changes observed can be mostly attributed to G2 cells as a) changes is S-phase cells mostly appeared due to increase at the S/G2 boundary of the signal histogram and this increase was in some cases ascribed to S-phase cells due to usage of rigid gating, and b) changed proportions of mitotic cells were not microscopically observed.

The results thus show that all the studied radiation doses activate G2 checkpoint arrest in SkBr3 cells. G2 checkpoint arrest is also to a small level induced by the 18 h-incubation with GNPs (0.4 μg/mL). The irradiation in the presence of GNPs then gives an additive effect. The examples of results for non-irradiated GNP(−) vs. GNP(+) cells and these cells exposed to 1 + 1 Gy dose are shown in Figure 12B. Though the increase of G2 cells in GNP(+) specimens was <5%, the systematic occurrence of this trend reinforces our suspicion that the cell subpopulation increased in GNP(+) specimens and was defined by higher nucleus volumes, repair focus numbers, and γH2AX/H3K9me3 tags on the nanoscale, consists by nature from G2 cells. The increased numbers of γH2AX/53BP1 foci in irradiated GNP(+) cells might thus reflect a higher DNA content of G2 cells than enhanced DSB damage induction by GNPs.

### 3.5. Acute Cytotoxicity and Effects on Mitochondria of Irradiation, GNPs and Combined Treatments

**Figure 13:**
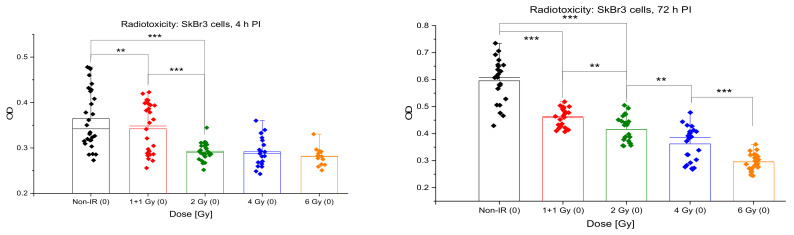
Effect of different γ-ray doses alone on SkBr3 cell viability as measured by MTT test. Dose-dependent survival is shown for 4 h (**left**) and 72 h PI (**right**), respectively. Four to six independent measurements for each irradiation dose are averaged and shown as the mean ± SD. Asterisks denote statistical significance of differences between indicated samples at *p* = 0.05 (*), *p* = 0.01 (**) or *p* = 0.001 (***). Two-tailed *t*-test, two-tailed *t*-test with Welsh correction or Mann–Whitney Rank Sum Test was used depending on the distribution normality of the analyzed variables.

The proportion of viable cells and their metabolic activity were measured for the indicated nanoparticle concentrations and radiation doses by MTT assay. As the reduction of MTT (tetrazolium salt (3-(4,5-dimethylthiazol-2-yl)-2,5-diphenyltetrazolium bromide; yellow) to formazan (purple/blue) depends on active mitochondria, MTT allowed us also to compare the treatment effects on these organelles. As mitochondria are energetic centers of the cell and the only cytoplasmic organelles containing DNA, they are potential alternative targets (in addition to the nuclear DNA) for the nanoparticle cytotoxicity and/or nanoparticle-enhanced radiotherapy. Figure 13 shows the results for the radiation dose-dependence for doses 0, 1 + 1, 2, 4 and 6 Gy, respectively, as measured at 4 h and 72 h PI. At 0.5 h PI, a nonlinear decrease of MTT values was observed in dependence of dose, with almost no effect at 1 + 1 Gy, followed by a significant decrease similar for all remaining 2, 4 and 6 Gy doses. With ongoing PI time, the MTT dose response approached a liner character, suggesting that MTT probably reflects more cellular factors at earlier post-irradiation periods, while more prominent cell dying (apoptotic and/or mitotic death) only appears and starts to dominate MTT measurements late after irradiation.

**Figure 14:**
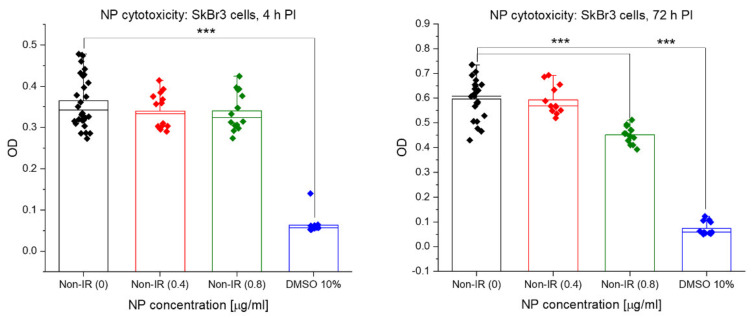
Cytotoxicity of GNPs by MTT test. Cell viability is measured in dependence of GNP concentration (0, 0.4 or 0.8 μg/mL) at 4 h (**left**) and 72 h PI (**right**), respectively. Post-irradiation times here, in non-irradiated samples, refer to the time points when the irradiated counterparts were evaluated. It means that the irradiated and non-irradiated samples were processed in the same way except of irradiation and analyzed in parallel. Four to six independent measurements for each irradiation dose are averaged and shown as the mean ± SD. Asterisks denote statistical significance of differences between indicated samples at *p* = 0.05 (*), *p* = 0.01 (**) or *p* = 0.001 (***). Two-tailed *t*-test, two-tailed *t*-test with Welsh correction or Mann–Whitney Rank Sum Test was used depending on the distribution normality of the analyzed variables.

**Figure 15:**
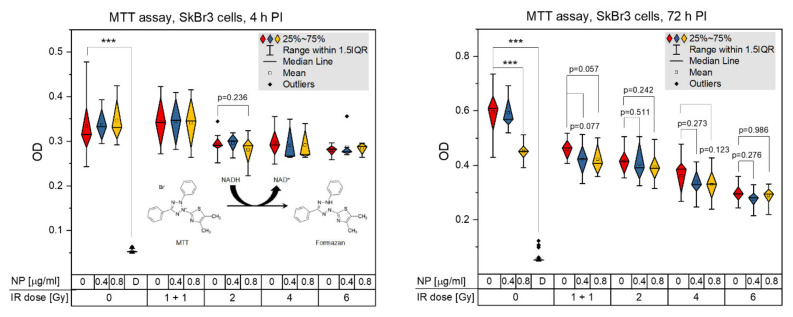
GNP-mediated radiosensitization of SkBr3 cells as evaluated in terms of MTT cell viability. MTT values are shown for cells exposed to different γ-ray doses in the absence or presence of 0.4 or 0.8 μg/mL GNPs and fixed at 4 h (**left**) or 72 h PI (**right**). Four to six independent measurements for each irradiation dose are averaged and shown as the mean ± SD. Rhombuses contain 50% of dataset values, which lay between the top (75%) and bottom (25%) quartiles. Asterisks denote statistical significance of differences between indicated samples at *p* = 0.05 (*), *p* = 0.01 (**) or *p* = 0.001 (***). Two-tailed *t*-test, two-tailed *t*-test with Welsh correction or Mann–Whitney Rank Sum Test was used depending on the distribution normality of the analyzed variables.

For the incubation period of 18 h and the concentration 0.4 μg/mL, cellular internalization of 10-nm GNPs had no effect on the mitochondria activity and cell viability measured by MTT, at either post-treatment period, i.e., 4 or 72 h after the end of incubation with GNPs (Figure 14). Similar results were also obtained for the higher (0.8 μg/mL) GNP concentration, where a marked cytotoxicity of GNPs was only recognized for the non-irradiated control but none of irradiated samples (Figure 14 and Figure 15). Combined irradiation in the presence of both GNP concentrations, applied 18 h before the exposure, slightly but systematically augmented the cell killing by radiation in terms of MTT results at all radiation doses (Figure 15). This enhancement of cell killing was absent at 4 h PI (Figure 15, left) but became more prominent though statistically insignificant at 72 h PI (Figure 15, right). Hence, comparison of specimens that were only irradiated (red rhombuses) to specimens irradiated in the presence of 0.4 μg/mL GNPs (blue rhombuses) points to a slight but insignificant radiosensitization effect of GNPs at all radiation doses. The results for the higher but still very low GNP concentration of 0.8 μg/mL (yellow rhombuses) did not further enhance the radiosensitization effect.

### 3.6. Clonogenic Cell Survival (Mitotic Death Analysis)

The results of the MTT test may suggest that SkBr3 cells can survive acute effects of irradiation but die later due to the mitotic death. Therefore, in the next step, we followed the survival and proliferation of SkBr3 cells in a longer-time perspective, using the clonogenic assay and real-time cell proliferation monitoring (xCELLigence).

**Figure 16:**
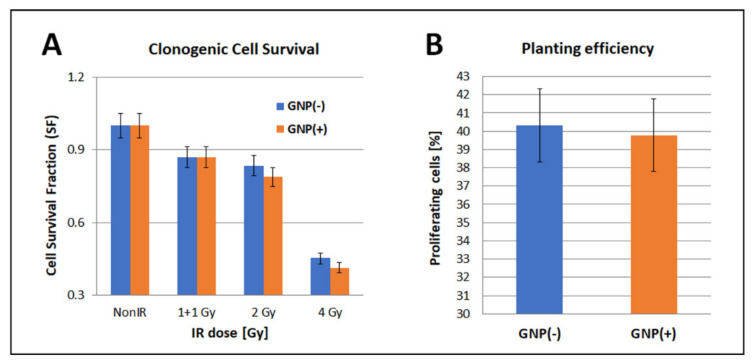
Clonogenic survival of SkBr3 cells upon exposure to increasing γ-ray doses in the presence (GNP(+)) or absence (GNP(−)) of 10-nm gold nanoparticles (Aurion, 0.4 μg/mL for 18 h). (**A**) Clonogenic survival fraction (SF) normalized to the plating efficiency. Blue bars—GNP(−) cells, orange bars—GNP(+) cells. (**B**) Influence of GNPs on plating efficiency of non-irradiated cells.

Clonogenic cell survival (mitotic death analysis) is the gold standard method in radiobiology to quantify radiation-induced cell death. This approach considers not only the cell dying immediately after irradiation due to apoptosis or necrosis, but also the delayed dying during the mitosis, due to the inability to separate chromosomes into the daughter cells (mitotic catastrophe). As the mitotic death is the most frequent type of cell death after exposure to moderate radiation doses, and in general for cancer cells, clonogenic assay reveal nanoparticle/RT effects not registered by MTT. As obvious from Figure 16, increasing radiation doses progressively decreased the clonogenic survival of SkBr3 cells, with a prominent drop observed for the 4 Gy dose (Figure 16A). A small difference was observed for 2 Gy doses delivered either as a single exposure or two 1 Gy fraction doses separated by 30 min. This suggests that 30 min separation between doses allows SkBr3 cells to partially recover from the radiation damage. The presence of GNPs (0.4 μg/mL for 18 h) did not reduce the plating efficiency of (non-irradiated) SkBr3 mammary carcinoma cells (Figure 16B) and only insignificantly decreased a long-term survival and proliferation capacity of these cells after irradiation (Figure 16A). More precisely, a very small difference (within the frame of SD/SE) in the clonogenic survival between cells irradiated in the absence or presence of GNPs was observed for 2 Gy and 4 Gy doses, while almost identical results were obtained for the 2 Gy dose split into two 1 Gy fractions (Figure 16A). Clonogenic assay thus did not reveal any cytotoxicity (MTT to some extent) of 10-nm GNPs and only minor radiosensitization effect mediated by these nanoparticles at the concentrations and the incubation period studied.

### 3.7. Real Time xCELLigence Adhesion/Proliferation Assay

xCELLigence cell adhesion assay was performed for the same treatments and conditions as the clonogenic assay. In accordance with the clonogenic survival results, the irradiated cells showed a slowed proliferation (with minor effect for the 1 + 1 dose). Additional slowing in the presence of GNPs was observed only for the 4 Gy specimen.

## 4. Discussion

Mammary tumors, as represented here by SkBr3 cells [64], are the cancer type with the highest incidence among cancer types in women. Importantly, most breast tumors are treated with IR; however, the treatment is often difficult due to close proximity of the tumor to the heart or other tissues. In addition, inherited BRCA1 or BRCA2 germline mutations may dramatically increase the risk of secondary tumors in “normal” tissue encompassing the tumor, which is also partly irradiated during radiotherapy. De-escalation of the therapeutic radiation dose with preservation of the therapeutic effect on the tumor may thus be an important safety improvement. On the other hand, radioresistance of mammary tumors [54] gives rise to disease progression in many patients [92], emphasizing the need for new specific radiosensitizers of tumor cells.

The application of GNPs in radiation therapy (for review, see [7,32]) was suggested many years ago. However, the results published for a variety of cell types, radiation qualities and nanoparticle types and sizes appear in some cases contradictive, pointing to critical and still unknown importance of these factors in response of nanoparticle-treated cells to irradiation [13,32]. Further research to gain a better understanding of this complex cellular response and the mechanisms behind the induced processes is stimulated in particular by the debate on the often conflicting outcomes of clonogenic survival experiments and analyses of DNA damage and repair using single-cell microscopy (see e.g., [13,14,31,32]). Some studies demonstrated increased clonogenic dying of irradiated cells due to nanoparticle-augmented DNA damage [93], while others reported increased cell dying without being accompanied by enhanced DNA damage [6] or vice versa (reviewed in [32]).

To make the situation even more complex, GNPs in numerous cell- or tissue-based studies lacked the ability to induce both adverse and acute toxicity effects and have been thus deemed biocompatible for biomedical applications [5,7,94]. However, in other experiments, a significant cytotoxicity has been demonstrated in terms of both enhanced cell dying and DNA damage [95] (reviewed in [96]). Absent or very low cytotoxicity was observed especially for nanoparticles approaching the size of GNPs used in the present work, i.e., 10 nm [97,98,99]. On the other hand, decreased MTT viability for ~20% was observed for hBMSCs and HuH-7 cells co-cultured with different concentrations of 15-nm or 30-nm GNPs [100]. Taggart et al. [101] then also revealed DNA damage in MDA-MB-231 human breast cancer cells after incubation with 1.9-nm GNPs; however, the subsequent irradiation did not cause any additional difference between GNP-treated and untreated cells. The literature [100,102] thus demonstrates a high complexity of nanoparticle effects in cells, emphasizing the importance of chemical and biological processes in this context, in addition to physical properties of nanoparticles [17].

To shed a new light on this confusing situation, we performed a comprehensive analysis of the cell viability and proliferation capacity, cell cycle distribution and DNA damage induction and repair. An extensive effort was dedicated to a multi-parametric analysis of DSB repair foci and chromatin architecture. Changes in the architecture of these structures can be observed after radiation DNA damage and during subsequent repair [37,40,56,62,103,104,105]. Molecular architecture/topology of repair foci and chromatin domains can be thus expected to reflect the character of DNA lesions and their repair mechanism, and, in turn, influence this mechanism as well as additional cellular processes [106]. For instance, the densely packed heterochromatin labeled with H3K9me3 antibodies has been shown to relax after DSB induction in order to allow access of repair proteins to damage sites and assembly of huge repair complexes (repair foci) [62,81,107]. This decondensation was dependent both on the dose and type of the incident radiation [56,62]. Evaluation of heterochromatin architecture and accessibility to H3K9me3 antibodies was therefore used in the present study as an additional indicator of potential differences between GNP-treated and untreated cells in DNA damage induction and repair as well as the overall cell status upon irradiation.

As a unique aspect of the present work, the induction and repair of DNA double strand breaks have been studied in parallel on the same samples at the micro- (Figure 1, Figure 2, Figure 3, Figure 4, Figure 5, Figure 6, Figure 7 and Figure 8) and nanoscale (Figure 9, Figure 10 and Figure 11) by high-resolution confocal microscopy and SMLM, respectively. Application of SMLM made it possible to compare even subtle differences between the architecture of repair foci and heterochromatin domains for different cell specimens, with a resolution of single molecules and 10 nm precision.

Numerous hypotheses have been postulated concerning the often-occurring discrepancy in the relationship between the reduction of the cell survival and enhanced DNA damage in cells irradiated in presence of nanoparticles. In the present manuscript, we used very low (0.4 μg/mL) GNP concentrations to avoid possible cytotoxicity. More significant cytotoxicity (MTT) has only been observed for twice as high GNP concentration (Figure 14) in non-irradiated cells. However, this drop in MTT viability in cells incubated with 0.8 μg/mL GNPs compared to 0.4 μg/mL GNP does not appear in either of irradiated specimens.

**Figure 17:**
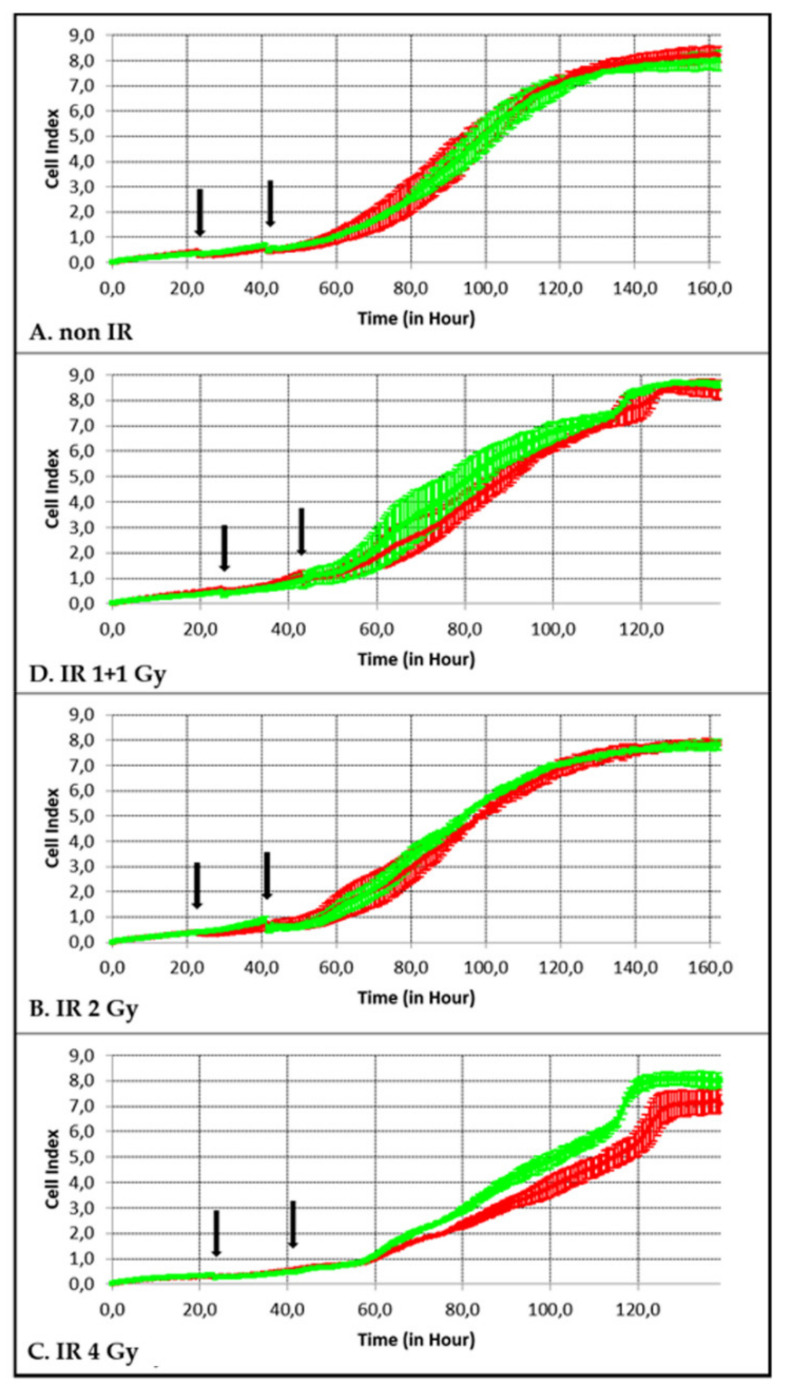
Real-time monitoring (xCELLigence RTCA) of SkBr3 cell adhesion after irradiation with the indicated doses of γ-rays in absence (**green**) or presence (**red**) of GNPs (18-h incubation with 0.4 μg/mL GNPs). The graphs show the cell adhesion as the mean cell index value from 2-well plates (±SD) plotted against post-irradiation time. Time point 0 denotes the point at which the E-plate was first scanned after cell addition. The graph shows the results for 4000 cells seeded at the beginning of the experiment, as this seeding density was identified as optimal. The time points of GNP addition and irradiation plus GNP removal are indicated by black arrows.

In contrast to absence of cytotoxicity in the non-irradiated cells (Figure 14, Figure 15, Figure 16 and Figure 17), irradiation in presence of GNPs mildly increased the average numbers of colocalized γH2AX and 53BP1 foci per nucleus, as followed from the evaluation of [γH2AX+ 53BP1] focus induction at the microscale (Figure 3(left) and Figure 5A) and γH2AX molecule clusters at the nanoscale (Figure 10). The slight γH2AX signal increase at the nanoscale (Figure 9, Figure 10 and Figure 11) is in concordance with our results obtained in former experiments [14]. Interestingly, a more detailed data inspection and micro-morphologic analysis of γH2AX and 53BP1 foci (Figure 7) revealed that the average number of repair foci per nucleus in GNP(+) cells does not grow due to a small but “systemic” increase of focus numbers over the whole cell population. It growths as the consequence of expansion of a cell subpopulation with very high focus numbers. The focus numbers in this subpopulation are comparable for GNP(+) and GNP(−) cells.

As G2 cells have twice the amount of DNA (2N) compared to G1 cells (1N), they also contain roughly twice the number of repair foci after irradiation [108]. A hypothesis can be thus postulated that even the very low (0.4 μg/mL) GNP concentration studied in the present manuscript does not augment radiation insertion of DSBs into the nuclear DNA (Figure 3, Figure 4 and Figure 5A) but enhance the G2/M checkpoint arrest and the proportion of SkBr3 cells at the G2 phase of the cell cycle (Figure 12). This possibility is further supported by larger average volumes of GNP(+) cell nuclei as compared to untreated cells (Figure 6). Flow cytometry subsequently provided direct evidence to this hypothesis, showing larger cell subpopulations in the G2 phase cells in GNP(+) specimens than GNP(−) ones (Figure 12). Importantly, GNPs stimulated the G2/M arrest not only in all irradiated specimens but also in non-irradiated controls (Figure 12). This demonstrates that the observed strengthening of the G2/M arrest is provoked by GNPs themselves. One possible mechanism responsible for the stronger G2/M arrest in GNP(+) cells could be the overexpression of GADD45 (Growth Arrest and DNA Damage-inducible 45) proteins [109], which have been implicated in regulation of many cellular functions including DNA repair, senescence, response to a genotoxic stress and, in parallel, cell cycle [109]. The G2/M arrest upon GNP-treatment has recently been observed also for another mammary cancer cell type, MCF-7 [110].

As G2 cells are considered the most sensitive to irradiation compared to cells in other phases of the cell cycle [111,112,113], some enhancement in radiation cell killing in presence of GNPs could be expected. Indeed, the increase in the proportion of cells with many γH2AX/53BP1 foci in GNP(+) cells relative to GNP(−) cells was noticeable at 1 + 1 and 2 Gy radiation doses but disappeared at the highest 4 Gy dose (Figure 7, third line). At the same time, in flow cytometric measurements (Figure 12), the total proportion of G2-phase cells significantly decreased after the 4 Gy exposure in both GNP(+) and GNP(−) cells. The difference between GNP(+) and GNP(−) specimens in extent of the G2 cell subpopulation was highest in non-irradiated cells and then decreased with dose. Finally, GNP(+) cells proliferated significantly less after irradiation than GNP(−) cells only after exposure to the highest dose of 4 Gy in real-time analyses of cell proliferation using xCELLigence system (Figure 17). A similar trend was also observed with clonogenic survival assay (Figure 16). Altogether, these results confirm that the cells with increased γH2AX/53BP1 focus numbers correspond to cells in the G2 cell cycle phase, which are more radiosensitive [111,112,113] and therefore significantly eliminated by radiation, with the efficiency growing with dose. The hypothesis proposed above is supported by an excellent agreement between the cell survival data and results of the repair focus analysis.

In contrast to proliferation assays, the extent of GNP-mediated radiosensitization approached statistically significant (*p* = 0.05) values only for the lowest (1 + 1 Gy) dose in MTT tests (Figure 15). This result is more difficult to explain. The differences of MTT values between GNP(+) and GNP(−) specimens were only small and thus susceptible to even faint influence. For instance, specific states of individual specimen cell populations may modify nanoparticle intake and/or GNP clump formation within cells [32]. Unfortunately, these phenomena cannot be directly quantified in the current study. In addition, mitochondria represent a putative target for GNP-mediated radiosensitization [101,114,115] but, at the same time, irradiation significantly enhances their biogenesis [116]. The dose-dependence of MTT values is thus not trivial and some studies challenged usage of this method in radiobiological research [116]. In any case, MTT has been reported to significantly underestimate real cell dying after irradiation [116].

As the number of electrons released from GNPs by radiation increase with radiation dose (until the maximum is reached), more high-energy electrons can theoretically reach the cell nucleus and generate more ROS along their trajectories, and in turn, cause more complex DNA damage. Hence, slightly increased occurrence of complex DSBs might be expected in both G1 and S/G2 cells. The increase in DSB complexity can be manifested as a more prominent persistence of DSBs at late (24 h) post-irradiation times but not necessarily as a higher maximum number of DSBs at the early times after irradiation. For the reduced ability of cells to repair complex DSB lesions completely, a small increase of γH2AX/53BP1 repair foci could be expected in the presence of GNPs. Consistent with this hypothesis, a slightly higher (*p* = 0.01) number of γH2AX/53BP1 foci persisted in SkBr3 cells irradiated in presence of GNPs with the 1 + 1 Gy or 2 Gy dose at 24 h PI (Figure 3, right). However, the greater persistence of repair foci in GNP(+) cells (Figure 3, right) probably does not significantly contribute to a radiosensitizing effect of GNPs. This statement is substantiated by statistically comparable numbers of persistent repair foci in GNP(−) and GNP(+) cells at the highest dose of 4 Gy (Figure 3, right), for which the largest difference in viability of GNP(−) and GNP(+) cells was observed (Figure 16 and Figure 17). The absent increase of persistent foci in GNP(+) cells at 4 Gy (Figure 3, right) can probably be explained by the dominant effect of irradiation alone at such a high dose.

Slightly enhanced generation of complex DNA lesions in irradiated GNP(+) cells compared to GNP(−) cells can be explained considering recent studies, in which small nanoparticle amounts were detected in the cell nucleus by Transmission Electron Microscopy (TEM) [117,118,119,120] or Raman Spectroscopy [121]. This can theoretically happen either due to disruption of the nuclear envelope by ROS (generated by nanoparticles in the cytoplasm) or during mitosis, when the envelope is disintegrated [122]. On the other hand, nanoparticles have been exclusively located to the cytoplasm in the extensive literature. In our previous papers [6,46], Synchrotron Radiation Deep UV (SR-DUV) microscopy [46], applied for the given purpose for the first time, showed that even ultra-small (3 nm) platinum and gadolinium nanoparticles are accumulated in the cytoplasm, especially within lysosomes. Hence, the cytoplasmic location can also be predicted for larger (10-nm) gold nanoparticles used in the present study. This expectation is also supported by our earlier TEM microscopy observations (unpublished but similar in conclusions, e.g., to [123]).

However, a limited presence of nanoparticles in the cell nucleus suggested by the above-indicated references is well compatible with our results showing a statistically significant but only slight increase in the number of difficult-to-repair (complex) DNA lesions in irradiated GNP(+) cells compared to GNP(−) controls.

A few GNPs in the nucleus could produce several clouds of spatially concentrated secondary electrons and ROS, which can generate several additional complex DNA lesions compared to GNP(−) controls. Alternatively, only long-live ROS produced by irradiated nanoparticles within the cytoplasm can reach the nucleus by diffusion, as secondary electrons are too limited in the range of their active pathway. Although research into the extent to which ROS formed in the cytoplasm can contribute to nuclear DNA damage is currently underway, it has been reported that cytoplasmic ROS can attack DNA [124]. Additional oxidative damage to the DNA bases and single-strand breaks (SSB) of the DNA [109], occasionally leading to DSBs if they occur in close proximity to each other, could be thus detected in GNP(+) cells compared to GNP(−) controls. However, ROS diffusing to the nucleus from the cytoplasm could be expected homogeneously dispersed when reaching the nucleus, in contrast to ROS concentrated along tracks of secondary electrons. Hence, an increase of SSBs and simple DSBs could be expected rather than an increase of complex DSBs. Moreover, some studies implicated inhibition of cellular antioxidants, such as thioredoxin reductase, to play a critical role in radiosensitization by metal nanoparticles [69,125]. Since SkBr3 cells used in the present work dispose with a high level of this enzyme due to intensive basal expression of the corresponding TrxR gene [67], the amount of cytoplasmic ROS and ROS-mediated DNA harm could be efficiently reduced in this cancer cell type.

Considering this different genetic background of cell types, it is not surprising that studies with different cell and nanoparticle types provided highly variable and frequently the opposite results on the influence of GNPs on DSB repair foci—their generation, repair kinetics and long-term persistence (see [5,6] and citations therein). It is therefore interesting that we did not observe increased radiation damage to DNA either in the current 10-nm GNP study or in our previous publications [6,13] with ultra-small (>3 nm) gadolinium and platinum nanoparticles. On the other hand, enhancement of the G2/M cell cycle arrest was observed only for 10-nm GNPs [6,13]. In accordance with this result, cell cycle arrest enhancement has been shown to depend on the nanoparticle size, with no effect registered for very small (<3 nm) nanoparticles in most studies (reviewed in [17]). In the context of the present work, these results provide further evidence that the increased average number of γH2AX/53BP1 repair foci per nucleus (Figure 3, left) has nothing to do with increased radiation damage to DNA in the presence of GNPs, but actually reflects the increase in G2 cell subpopulation due to enhanced G2/M cell cycle arrest. In general, the outlined results confirm that nanoparticle-mediated cell effects are strongly dependent on nanoparticle properties, as discussed later.

Another interesting and seemingly paradoxical phenomenon recognized in the present study is a faster DSB repair kinetics in SkBr3 cells irradiated in presence of low GNP concentrations than in controls irradiated without GNPs. With the exception of the 4 Gy dose, this faster repair in GNP(+) cells (Figure 4) eventually ended with slightly higher numbers of persistent γH2AX/53BP1 foci at 24 h PI (Figure 3(left) and Figure 4), as already discussed. Thus, the hypothesis that low GNP concentrations do not significantly worsen radiation damage to DNA but, on the contrary, stimulate DSB repair, can be postulated.

Surprisingly, this behavior is not unprecedented in radiobiology, where, for instance, a low-dose exposure is known to improve DSB repair and the overall cell response to subsequently applied higher dose [126]. In addition, GNPs have been shown to shift the balance between the fast but less precise NHEJ (nonhomologous end joining) and the slow but precise HR (homologous recombination). The expression of many repair proteins involved in HR (e.g., RAD51, BRCA1, ATM, MRE11, RPA1, CHEK2, PCNA) has been down-regulated in the presence of nanoparticles in many studies [127] (reviewed in [17]) while downregulation of NHEJ components (e.g., Ku70 and AKT) has been reported much less frequently [128,129]. Lim et al. [130] demonstrated that DNA-PKcs—a critical component of the NHEJ repair pathway—is predominantly responsible for repair of DNA damage induced by (silver) nanoparticles. Both absence of the DNA-PKcs activity in DNA-PKcs-deficient cells and its chemical inhibition with NU7026 lead to a more prominent dose-dependent decrease in viability of the nanoparticle-treated cells after irradiation, as compared to controls without nanoparticles. The deficiency or inhibition of DNA-PKcs rendered the nanoparticle-treated cells with higher susceptibility to DNA damage and genome instability, which, in turn, contributed to more pronounced cell cycle arrest and cell death [131]. Interestingly, this combination of nanoparticle effects on cells is perfectly compatible with our current observation of a faster DSB repair and, at the same time, a stronger G2/M cell cycle arrest in SkBr3 cells irradiated in presence of GNPs.

This combined effect of GNPs on DSB repair and cell cycle could be explained, e.g., the downregulation of CHEK2, also observed in nanoparticle-treated cells. Through a cascade of events, CHEK2 activates the G1/S cell cycle checkpoint [132] and via its interaction with BRCA1 stimulates HR [133]. Downregulation of CHEK2 thus enhances the proportion of G2 cells (which are partly dependent on HR in contrast to G1 cells fully relying on NHEJ) and, at the same time, decreases the HR capacity. NHEJ could thus become the dominant repair pathway in GNP(+) cells. This is accompanied with a faster repair kinetics but slightly higher persistence of unrepaired DSBs (Figure 4) as NHEJ is inefficient in repairing some DSB lesion types, including complex DSBs (reviewed in [40]). These results further support the hypothesis that the slightly increased persistence of γH2AX/53BP1 foci at 24 h PI, observed in GNP(+) cells in the present study, occurs due to enhanced generation of additional complex DSB lesions in GNP(+) cells and/or slightly lower ability of these cells to repair particular DSB types.

The possibility of a faster DSB repair in irradiated cells treated with low GNP concentrations has been supported even more directly. In their study, Turnbull et al. [124] analytically quantified intracellular concentrations of GNPs using synchrotron X-ray fluorescence (S-XRF) microscopy. The study showed that while high GNP concentrations sensitized cells to irradiation, as expected, low quantities of the same nanoparticles induced the opposite effect, i.e., desensitized the GNP(+) cells compared to controls receiving radiation alone. The authors explained this observation, perfectly supporting the results of the present study, in two possible ways. Low concentrations of nanoparticles condition the cells with a stress–response, which decreases their sensitivity to subsequent irradiation. Alternatively, radiosensitive (G2) cells could uptake nanoparticles more efficiently than radioresistant cells [124].

In addition to directly measured faster disappearance of repair foci in GNP(+) cells, the hypothesis that low nanoparticle concentrations may accelerate DSB repair is reasoned also by the present analyses of chromatin architecture in GNP(−) and GNP(+) cells at the nanoscale. Despite the similar median values, SMLM analysis of chromatin architecture at the molecular level showed a higher dispersion of H3K9me3 antibody counts in GNP(+) compared to GNP(−) cells in two of three cell specimens analyzed (specifically in the non-irradiated specimen and the specimen exposed to 2 Gy) (Figure 10B). This observation demonstrates an increased heterochromatin accessibility for H3K9me3 antibodies and thus heterochromatin decondensation. This decondensation, in turn, points to an upregulated repair activity due to unconstraint repair protein trafficking. The trend towards a more extensive (hetero)chromatin decondensation, predicted here just on the basis of a greater data variability (which can be attributed to very low GNP concentrations used), should be verified for higher GNP concentrations, for which a more pronounced effect could be expected. Nevertheless, chromatin rearrangements could clearly be found between the specimens with and without GNPs (Figure 9, Figure 10 and Figure 11); thereby the width and position of the heterochromatin cluster peak remained equal, which agrees with findings described in [40] for the same cell line (Figure 11). However, the slope of the distance frequency curves was increasing with the dose applied at higher distances above the cluster size (Figure 11). This indicates an increase in randomness for these distances and thus an increase in relaxation of heterochromatin accompanied by the opening of the “heterochromatin barrier” for molecular trafficking required for repair activity.

The last factors that can dramatically affect the presented results, which will be discussed here due to space constraints, are the energy of the incident radiation and nanoparticle composition. While MeV γ-rays are often used in clinical practice, only few experimental studies have been performed with radiation energies at this range [94], partly due to theoretical predictions of insignificant effects (reviewed in [7,94]). These experiments showed that the radiosensitizing effect sensitively depends on the combination of nanoparticle material and radiation energy. Irradiation of metal nanoparticles with monochromatic synchrotron X-rays with an energy corresponding to or exceeding the K-edge energy of the particular element showed very promising radiosensitizing ratios [94]. However, while gadolinium-based nanoparticles ensured complete tumor cell destruction upon irradiation of tumor spheroids with monochromatic synchrotron X-rays of 50.25 keV, the effect disappeared at a very close energy of 50.0 keV (reviewed in [94]). These results demonstrate that precise tuning of the incident radiation energy may be a critical factor influencing the intensity of metal nanoparticle-mediated radiosensitization. Simulations suggested that the extent of GNP-mediated radiation sensitization should be significantly higher for keV photons than photons in the MeV range [134,135,136]. This conclusion was proved also by some experimental studies [27]. For instance, the dose enhancement for different gold and bismuth nanoparticle sizes and concentrations descended with increasing radiation energy as follows: 95.3 keV synchrotron beam (16–32%), 150 kVp superficial beam (12–21%), and 6 MeV beam (2–5%) [137].

High (MeV) energies of both γ-rays and X-rays used in the present manuscript together with very low GNP concentrations may be thus a critical combination of experimental conditions explaining why only weak (insignificant) enhancement of radiation DNA damage in GNP(+) cells and/or radiation killing of these cells was observed (reviewed in [94]). However, in several studies, low GNP concentrations exerted a marked radiosensitization even upon cells’ exposure to MeV clinical beams [138]. For example, the dose enhancement ratios for GNPs exposed to 6 MV Linac beams (i.e., the radiation used in the present study) ranged from 14–287%, depending on the nanoparticle size (10–100 nm) [139]. The radiosensitization was more prominent when the flattening filter of the linear accelerator was removed [140] as it has been carried out in the present manuscript. However, the specific intracellular concentrations of GNPs in [139] were not determined, which precludes direct comparison of the dose enhancement with theoretical simulations.

Cytotoxic and radiosensitizing effects of a particular GNP type were explored in detail in the present study. However, a tremendous range of nanoparticle materials, material combinations, sizes, shapes, coatings and surface modifications with different molecules dramatically influences specific nanoparticle behavior, such as cell internalization and intracellular localization, EPR effect, affinity to specific tumor cells, ROS production, etc. On the one hand, this multifactorial variability offers a possibility of targeted combinatorial design of therapeutic nanoparticles. On the other hand, it seriously complicates both experimental and theoretical examination of nanoparticle effects on cells prior to and after irradiation. Better understanding of such complicated systems [5] can be brought about by a quantitative multiscale modeling approach, which is under development and will allow joint modeling of physical, chemical and biological post-irradiation processes [139,141,142]. To generate data for this modeling, experimental studies examining multiple biological effects in detail exerted by a given nanoparticle type in a particular cell type, as exemplified by the current study, are necessary, in addition to more frequent studies comparing only a few selected effects across several nanoparticle and cell types. A critical factor that should be quantitatively determined in all future studies, if possible, is the number of nanoparticles internalized in single cells. Correlating these levels to treatment (NP, IR, NP plus IR) responses of individual cells would be a breakthrough in nanoparticle research [32].

Single molecule localization microscopy (SMLM) has been proven to be a very important method in this context as it offers quantitative data on selected molecule distribution patterns in individual cells, without a need for complicated and sometimes misleading image analysis [143]. Under some circumstances, non-labeled nanoparticles can directly be studied by SMLM [28]. Through this paper, SMLM data published by our consortium so far [13,14] were supported and elucidated in more detail. However, the breakthrough towards a conclusive mechanism solving the contradictions found in the literature will require further investigations, not only experimental, but also appropriate theoretical simulations (see chapter 2 and 3 in [32]) and meta-analyses, as already outlined.

To summarize, the complex data obtained in the present work suggest that GNPs, even in very low concentrations, radiosensitize tumor cells by increasing the proportion of more radiosensitive G2 cells. Studies providing similar results on cell cycle arrest enhancement for other cell and nanoparticle types [110,144,145] support a more general relevance of our observations obtained for SkBr3 cells. While increased DSB damage in the presence of nanoparticles has been traditionally considered the mechanism responsible for nanoparticle-mediated radiosensitization, the extent of the DSB induction by clinically relevant γ-ray doses remained uninfluenced by both herein examined GNPs and gadolinium or platinum nanoparticles studied previously [6,13,46]. On the other hand, the number of persistent (complex) DSBs was slightly elevated by GNPs in the present study, without any obvious effect on post-irradiation cell survival. Based on the data for a particular GNP type explored here, we cannot exclude that persistent DSBs and their negative effect on cell survival could become more prominent at higher nanoparticle concentrations or with different NPs. However, our previous studies with 3-nm gadolinium and platinum nanoparticles do not favor this possibility [6,13,46]. Finally, DSB repair seems to be accelerated in cells with low GNP concentrations, though on the expenses of lower repair precision.

Hence, with a global perspective, we incline to the conclusion that the current, purely physical hypothesis on the mechanism of metal nanoparticle-mediated radiosensitization—based on emission of secondary electron showers by irradiated nanoparticles, local production of ROS clouds and consequently enhanced DNA damage—cannot fully explain what is observed in ours and other published experiments. Indeed, generated ROS and decreased antioxidant capacity of cells may not only alter cell signaling, as reported here, but also harm cytoplasmic structures critically involved in cell survival, such as lysosomes [6,13], mitochondria [101,114,115] and/or endoplasmic reticulum. Nanoparticle-mediated cell effects disclosed in the present manuscript could thus act in an additive or even synergic manner with numerous radiosensitizing mechanisms described elsewhere [6,13,101,114,115]. Testing of these individual radiosensitization mechanisms and their dependence on complex radiation-nanoparticle-cell type systems is an ongoing goal for future research. Our current and previous results, together with extensive literature, thus draw a complex picture of metal nanoparticle-mediated radiosensitization, as recently reviewed, e.g., in Penninckx et al. [17] or Tremi et al. [94].

## 5. Conclusions

In contrast to several literature contributions, the present study combines a detailed analysis of DNA damage and response at the microscale (confocal microscopy) and nanoscale (SMLM) level with analyses of various aspects of cell viability on the same samples and under the same conditions. We show that the application of even very low concentrations of GNPs prior to irradiation increases the proportion of SkBr3 cells with higher numbers of colocalized γH2AX and 53BP1 foci and, to a minor extent, the number of persistent DSBs. The cells with increased repair focus numbers seem to correspond with the more radiosensitive cells in the G2-phase of the cell cycle. A slightly decreased viability can be measured for SkBr3 GNP(+) cells irradiated in presence of GNPs, although the higher radiation damage to DNA appears to be compensated by increased, GNP-stimulated repair activity of SkBr3 cancer cells. Hence, a slightly decreased post-irradiation survival could be ascribed to occasional occurrence of complex DSBs, enhanced by GNPs. More prominent effects could be observed for higher GNP concentrations and/or lower energies of ionizing radiation.

## Data Availability

Data are part of the IBP and KIP SMLM data archive and can be obtained upon request to the corresponding author.

## Supplementary Materials

The following supporting information can be downloaded at: https://www.mdpi.com/article/10.3390/pharmaceutics14010166/s1, Figure S1: Examples of raw data of the immunofluorescent detection of γH2AX (green) and 53BP1 (red) foci in SkBr3 cells incubated with 0.4 μg/ml GNPs for 18 h prior to irradiation with a single γ-ray dose of 2 Gy and left repair DNA damage for 0.5, 1, 8 and 24 h post irradiation (PI); Figure S2: Specificity of anti-γH2AX and anti-53BP1 antibodies proved by Western blotting.
