# Supplementary material for: Incorporation of Low Concentrations of Gold Nanoparticles: Complex Effects on Radiation Response and Fate of Cancer Cells
Source: Pharmaceutics. 2022 Jan 11;14(1):166. doi: 10.3390/pharmaceutics14010166 (PMC8781406; doi:10.3390/pharmaceutics14010166)
Supplement: Supplementary file 1 [file pharmaceutics-14-00166-s001.zip › pharmaceutics-1472226-supplementary.pdf]

pharmaceutics

MDPI

# Supplementary Materials: Incorporation of Low Concentrations of Gold Nanoparticles: Complex Effects on Radiation Response and Fate of Cancer Cells

Lucie Dobešová , Theresa Gier , Olga Kopečná , Eva Pagáčová , Tomáš Vičar , Felix Bestvater , Jiří Toufar , Alena Bačíková , Pavel Kopel , Radek Fedr , Georg Hildenbrand , Iva Falková , Martin Falk and Michael Hausmann

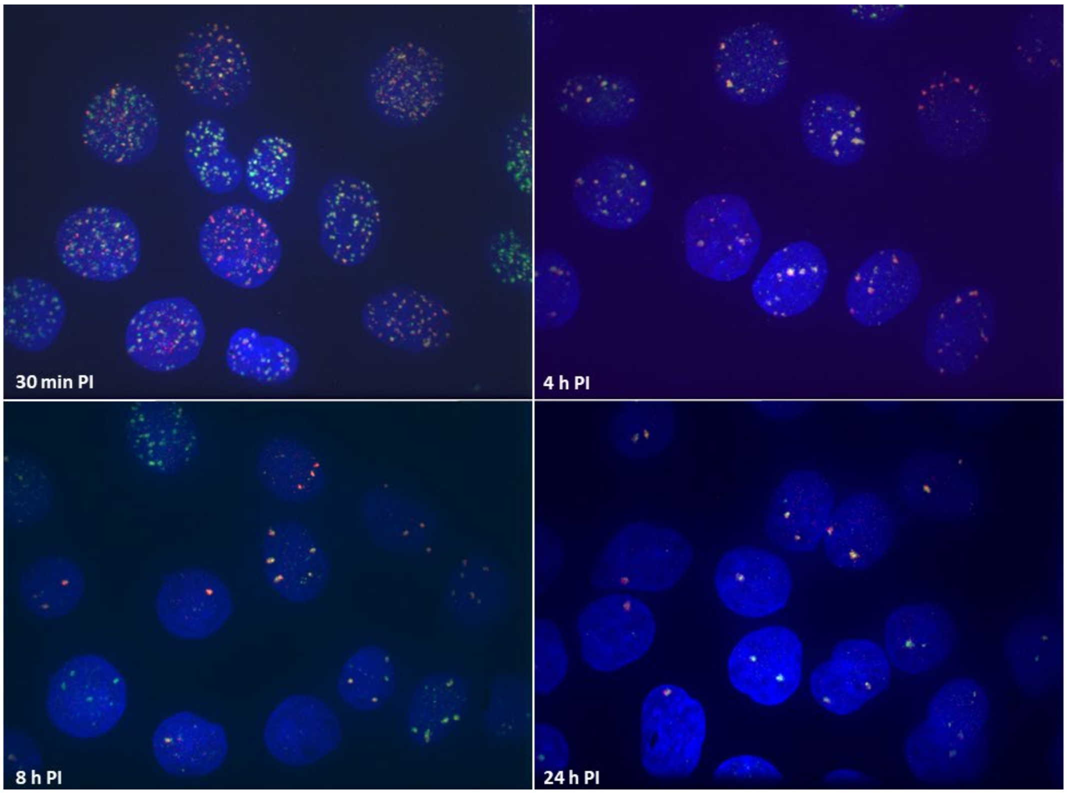


**Figure S1.** Examples of raw data of the immunofluorescent detection of γH2AX (green) and 53BP1 (red) foci in SkBr3 cells incubated with 0.4 µg/ml GNPs for 18 h prior to irradiation with a single γ-ray dose of 2 Gy and left repair DNA damage for 0.5, 1, 8 and 24 h post irradiation (PI). Chromatin is counterstained with DAPI (blue). .

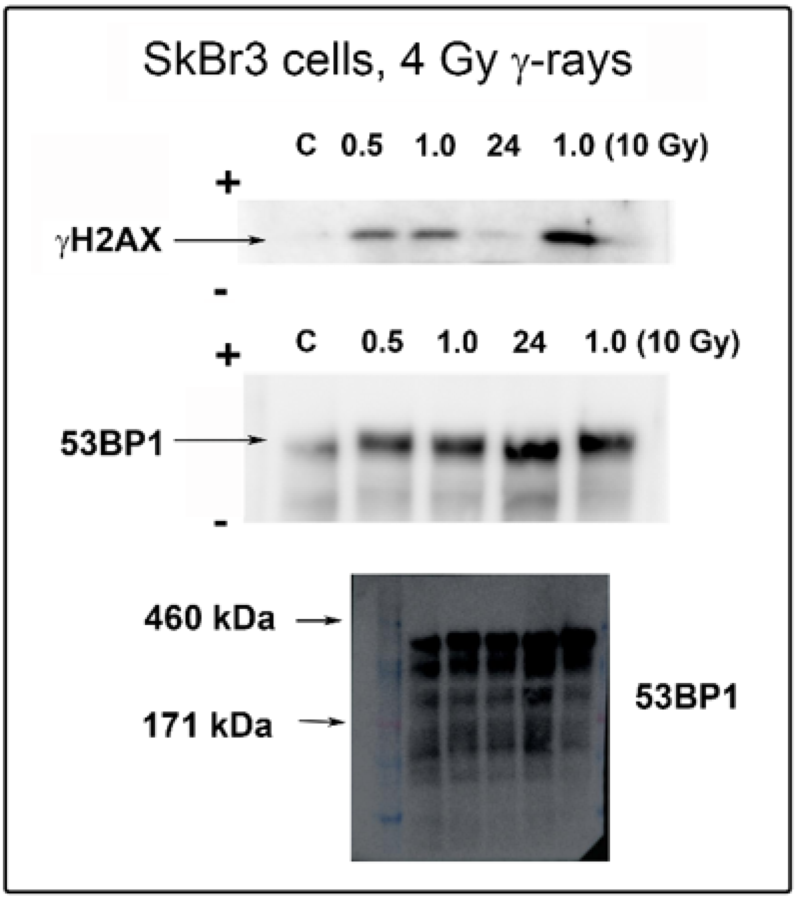


**Figure S2.** Specificity of anti-γH2AX and anti-53BP1 antibodies proved by Western blotting. Specific bands of γH2AX (top) and 53BP1 (middle) can be seen for SkBr3 cell specimens irradiated with 4 Gy γ-rays and fixed at the indicated periods of post-irradiation time. 10 Gy sample fixed at 1 h PI served as a high-dose control. The bottom gel shows the position of the large 53BP1 protein relative to a size marker. Raw images are shown prior to the result normalization for the reference protein (see Methods for details).
